# Artificial Intelligence in Predicting Postoperative Nausea and Vomiting Among Surgical Patients Following General Anesthesia: A Systematic Review

**DOI:** 10.7759/cureus.115542

**Published:** 2026-08-31

**Authors:** Vendhan Ramanujam, Samyr Carneiro, Kumaran Ramanujam, Amy Arthur, Eden Addisu, Shilpa N Chouhan

**Affiliations:** 1 Department of Anesthesiology, The Warren Alpert Medical School of Brown University, Providence, USA; 2 Department of Global Tech Platforms, Walmart Inc, Sunnyvale, USA; 3 Department of Anesthesiology, Jefferson Einstein Philadelphia Hospital, Philadelphia, USA

**Keywords:** artificial intelligence, general anesthesia, machine learning, perioperative outcomes, risk prediction

## Abstract

Artificial intelligence (AI) may improve perioperative risk prediction by integrating complex clinical and physiological data, but its performance and readiness for clinical use remain uncertain. We evaluated AI models predicting postoperative nausea and vomiting (PONV), perioperative hypotension, prolonged intensive care unit (ICU) stay, in-hospital mortality, and neurological complications after general anesthesia. We searched bibliographic databases from inception to June 2026 for studies developing or validating AI-based prediction models in surgical patients undergoing general anesthesia. Two reviewers independently screened studies, extracted data, and assessed risk of bias and applicability using PROBAST+AI. Owing to heterogeneity in populations, outcomes, prediction horizons, algorithms, and validation methods, findings were synthesized narratively. Of 2,329 records identified, 22 studies were included. Sample sizes ranged from 221 to 106,860 participants. PONV models had area under the receiver operating characteristic curve (AUC) values of 0.561-0.960, whereas hypotension models ranged from 0.630 to 0.950. Models for prolonged ICU stay, mortality, and neurological complications showed AUCs up to 0.920, 0.930, and 0.890, respectively. Higher AUCs were not consistently accompanied by balanced sensitivity and specificity. All studies had high overall risk of bias, principally because of limited external validation, incomplete calibration reporting, and analytical concerns. AI shows promise for perioperative risk prediction, particularly for short-horizon hypotension and data-rich postoperative outcomes. However, substantial heterogeneity, high risk of bias, and scarce external validation preclude routine clinical implementation. Prospective, multicenter, calibrated, and impact-evaluated models are required.

## Introduction and background

General anesthesia enables millions of surgical procedures to be performed every year, but the perioperative period remains vulnerable to adverse physiological and clinical events from general anesthesia that vary in timing, severity, and mechanism. These events may cause substantial patient discomfort, delay mobilization and discharge, necessitate escalation of monitoring or treatment, increase resource utilization, and, in severe cases, contribute to persistent disability or death. Postoperative nausea and vomiting (PONV) is one of the common complications after general anesthesia that can affect nearly 30% of all surgical patients and stretch its burden across their recovery, satisfaction, outcomes and hospital finances [1]. Its risk involves multiple factors, and if they are missed and not treated with adequate precautions and medications, it can trigger a cascade of clinical breakdowns. Accurate estimation of individual risk is therefore central to perioperative planning, selection of preventive measures, intraoperative vigilance, postoperative disposition, and communication with patients and multidisciplinary teams [2].

Risk prediction in anesthesia has traditionally relied on clinical judgment, physiological thresholds, and parsimonious scores developed from a restricted set of prespecified variables. For PONV, risk is commonly estimated using combinations of patient-specific factors (female sex, younger adults, non-smoking status, previous PONV or motion sickness), anesthesia-related factors (pre- or intraoperative dehydration, use of volatile anesthetics, nitrous oxide and opioids), and surgical factors (prolonged surgical duration, laparoscopic surgeries, neurosurgery, ear, nose, and throat surgeries, gynecological surgeries) [3]. Although there are risk assessment tools that are practical to use, the occurrence of perioperative adverse events is shaped by complex biological interactions among baseline characteristics, comorbidities, laboratory measurements, surgical procedures, anesthetic techniques, medications, physiological trajectories, and postoperative treatment. The relevance of these predictors may also change over time: preoperative variables characterize baseline susceptibility, intraoperative signals capture evolving physiological stress, and early postoperative data may indicate failure to recover as expected. Conventional models may have limited ability to represent nonlinear associations, higher-order interactions, time-varying measurements, and the large number of variables that are routinely recorded in electronic health records and anesthesia information management systems [2,4]. Unlike simple clinical risk scores that generally combine a limited number of predefined predictors into a static estimate, artificial intelligence (AI)-based models can integrate larger numbers of heterogeneous and potentially time-varying variables, allowing them to capture nonlinear relationships and interactions that may be difficult to represent using conventional approaches. This capacity may be particularly relevant in perioperative care, where patient characteristics, physiological measurements, anesthetic exposures, and postoperative trajectories evolve over time.

AI, particularly machine learning and deep learning, offers a data-driven approach to integrating these heterogeneous perioperative data. Algorithms such as decision trees, random forests, support vector machines, k-nearest-neighbor methods, gradient-boosting machines, artificial neural networks, recurrent neural networks, and ensemble models can learn complex relationships between combinations of predictors and subsequent clinical events [2,4]. In PONV research, these methods have incorporated the established risk factors together with age, body mass index, surgical type and duration, anesthetic and opioid exposure, antiemetic prophylaxis, postoperative pain, and patient-controlled analgesia-related variables [5-7]. For hypotension, models have used preoperative characteristics, induction medications, intraoperative vital signs, and high-fidelity arterial pressure waveforms to estimate the probability of an impending or early postoperative hypotensive episode [8-11]. Broader perioperative platforms have combined demographic, socioeconomic, comorbidity, medication, laboratory, procedural, and continuously measured physiological data to predict prolonged intensive care unit (ICU) stay, neurological complications, and in-hospital mortality [12,13]. These applications illustrate transition from a static, single-time-point risk assessment to a dynamic prediction across the surgical pathway.

Nevertheless, reported predictive performance varies substantially across outcomes, algorithms, populations, and clinical settings. Apparent differences in discrimination, sensitivity, and specificity may arise from variation in outcome definitions, assessment windows, surgical case mix, predictor availability, prediction timing, population representation, handling of missing data and class imbalance, feature selection, model tuning, and validation methods. Models derived from large retrospective datasets may achieve strong internal performance yet remain susceptible to overfitting, dataset shift, treatment-related confounding, and reduced transportability when applied to other hospitals or patient groups. Clinical value also depends on whether predictions are available at a time when management can be modified, whether the model maintains adequate sensitivity without generating excessive false alerts, and whether its outputs are interpretable and compatible with perioperative workflows [2,4,12,13]. This systematic review therefore evaluated AI-based models used to predict PONV as a primary outcome and other secondary outcomes that are interlinked through shared pathophysiology among surgical patients undergoing general anesthesia.

## Review

Methods

Study Design and Reporting

This systematic review was conducted and reported in accordance with the Preferred Reporting Items for Systematic Reviews and Meta-Analyses (PRISMA) 2020 statement [14]. Reporting was additionally informed by the Transparent Reporting of Multivariable Prediction Models for Individual Prognosis or Diagnosis-Systematic Reviews and Meta-Analyses (TRIPOD-SRMA) guideline, which is specifically intended for systematic reviews of prediction-model studies [15]. The review protocol was prospectively registered in PROSPERO (registration number: CRD420261435120).

Eligibility Criteria

The eligibility criteria were defined according to the key domains recommended for systematic reviews of prediction-model studies, including the target population, index model, predicted outcomes, prediction timing, clinical setting, and study design. The inclusion and exclusion criteria are presented in Table 1.

**Table 1:** Inclusion and exclusion criteria. AI, artificial intelligence

Criterion	Inclusion criteria	Exclusion criteria
Population	Surgical patients undergoing general anesthesia, irrespective of age, sex, surgical specialty, inpatient or ambulatory status, or elective or emergency status.	None
Mixed anesthesia populations	Studies with mixed anesthesia populations were eligible when data for patients receiving general anesthesia could be identified separately or when general anesthesia constituted the clearly defined target setting of the prediction model.	None
Prediction model	Studies developing, internally validating, externally validating, updating, or evaluating at least one multivariable AI or machine-learning model intended to predict an eligible perioperative outcome at the individual-patient level.	None
AI and machine-learning approaches	Artificial neural networks, deep neural networks, multilayer perceptrons, recurrent neural networks, long short-term memory networks, decision trees, random forests, gradient-boosting methods, extreme gradient boosting, LightGBM, CatBoost, support vector machines, k-nearest-neighbor algorithms, naïve Bayesian classifiers, Bayesian additive regression trees, and ensemble-learning methods were eligible.	None
Predictor timing	Models using preoperative, intraoperative, postoperative, or combined perioperative information were eligible.	None
Comparator	Studies were eligible irrespective of whether the AI model was compared with another machine-learning algorithm, a conventional regression model, an established clinical risk score, or usual clinical assessment. A comparator was not mandatory.	None
Outcomes	Studies predicting at least one prespecified outcome were eligible: postoperative nausea and vomiting as the primary outcome; perioperative hypotension, prolonged intensive care unit stay, in-hospital mortality, or postoperative neurological complications as secondary outcomes.	None
Study design	Prospective and retrospective cohort studies, observational model-development or validation studies, mixed-method studies containing an eligible prediction-model evaluation, and clinical trials reporting development or performance of an eligible prediction model.	Reviews, editorials, commentaries, protocols, case reports, non-human studies, purely technical or simulation studies without patient-level clinical data, and conference abstracts without sufficient methodological or performance information.
Predictive performance	Studies reporting at least one measure of predictive performance, such as area under the receiver operating characteristic curve, sensitivity, or specificity.	None
AI application	AI-based approaches predicting one of the prespecified clinical outcomes were eligible.	Studies using AI solely for image segmentation, automated monitoring, treatment recommendation, or signal processing without predicting one of the prespecified clinical outcomes.

Information Sources and Search Strategy

A systematic search was conducted in MEDLINE/PubMed, Embase, Scopus, Web of Science Core Collection, and Cochrane CENTRAL from database inception to June 30, 2026. The search combined controlled vocabulary and free-text terms relating to AI and prediction methods, including “artificial intelligence”, “machine learning”, “deep learning”, “neural network”, “random forest”, “decision tree”, “support vector machine”, “gradient boosting”, “XGBoost”, “LightGBM”, “k-nearest neighbour”, “Bayesian”, and “ensemble learning”; and perioperative setting, including “surgery”, “surgical”, “perioperative”, “intraoperative”, “postoperative”, “general anesthesia”, and “general anaesthesia”.

Terms relating to prediction, prognostic modelling, risk estimation, classification, model development, and validation were also incorporated. Search terms were adapted to the syntax and indexing system of each database. The complete search strategy for all databases is provided in Table 2. No language restrictions were applied. Reference lists of included studies and relevant reviews were screened to identify additional eligible reports.

**Table 2:** Search strategy.

Data base	Terms
PubMed	("Artificial Intelligence"[MeSH Terms] OR "Machine Learning"[MeSH Terms] OR "Deep Learning"[MeSH Terms] OR "Neural Networks, Computer"[MeSH Terms] OR "Data Mining"[MeSH Terms] OR "Decision Trees"[MeSH Terms] OR "Algorithms"[MeSH Terms] OR "Ensemble Learning"[MeSH Terms] OR "Prediction Methods, Machine"[MeSH Terms] OR "Predictive Learning Models"[MeSH Terms] OR "Pattern Analysis, Machine"[MeSH Terms] OR "Intelligent Systems"[MeSH Terms] OR "artificial intelligence"[Title/Abstract] OR "artificial-intelligence"[Title/Abstract] OR AI[Title/Abstract] OR "machine intelligence"[Title/Abstract] OR "computational intelligence"[Title/Abstract] OR "machine learning"[Title/Abstract] OR "machine-learning"[Title/Abstract] OR ML[Title/Abstract] OR "deep learning"[Title/Abstract] OR "deep-learning"[Title/Abstract] OR "representation learning"[Title/Abstract] OR "supervised learning"[Title/Abstract] OR "unsupervised learning"[Title/Abstract] OR "semi-supervised learning"[Title/Abstract] OR "reinforcement learning"[Title/Abstract] OR "transfer learning"[Title/Abstract] OR "federated learning"[Title/Abstract] OR "statistical learning"[Title/Abstract] OR "automated learning"[Title/Abstract] OR "predictive analytics"[Title/Abstract] OR "data mining"[Title/Abstract] OR "pattern recognition"[Title/Abstract] OR "pattern analysis"[Title/Abstract] OR "expert system*"[Title/Abstract] OR "knowledge-based system*"[Title/Abstract] OR "intelligent system*"[Title/Abstract] OR "clinical decision support system*"[Title/Abstract] OR "artificial neural network*"[Title/Abstract] OR "neural network*"[Title/Abstract] OR ANN[Title/Abstract] OR DNN[Title/Abstract] OR CNN[Title/Abstract] OR RNN[Title/Abstract] OR LSTM[Title/Abstract] OR GRU[Title/Abstract] OR "deep neural network*"[Title/Abstract] OR "convolutional neural network*"[Title/Abstract] OR "recurrent neural network*"[Title/Abstract] OR "long short-term memory"[Title/Abstract] OR "long short term memory"[Title/Abstract] OR "gated recurrent unit*"[Title/Abstract] OR "multilayer perceptron*"[Title/Abstract] OR "multi-layer perceptron*"[Title/Abstract] OR MLP[Title/Abstract] OR "feedforward neural network*"[Title/Abstract] OR "feed-forward neural network*"[Title/Abstract] OR "backpropagation neural network*"[Title/Abstract] OR "back-propagation neural network*"[Title/Abstract] OR "probabilistic neural network*"[Title/Abstract] OR "self-organizing map*"[Title/Abstract] OR "self organising map*"[Title/Abstract] OR "decision tree*"[Title/Abstract] OR "classification tree*"[Title/Abstract] OR "regression tree*"[Title/Abstract] OR CART[Title/Abstract] OR "C4.5"[Title/Abstract] OR "C5.0"[Title/Abstract] OR "random forest*"[Title/Abstract] OR "balanced random forest*"[Title/Abstract] OR "conditional random forest*"[Title/Abstract] OR "conditional inference forest*"[Title/Abstract] OR cforest[Title/Abstract] OR "extremely randomized tree*"[Title/Abstract] OR "extremely randomised tree*"[Title/Abstract] OR "extra tree*"[Title/Abstract] OR "gradient boosting"[Title/Abstract] OR "gradient-boosting"[Title/Abstract] OR "gradient boosting machine*"[Title/Abstract] OR GBM[Title/Abstract] OR GBDT[Title/Abstract] OR "gradient boosting decision tree*"[Title/Abstract] OR "extreme gradient boosting"[Title/Abstract] OR XGBoost[Title/Abstract] OR LightGBM[Title/Abstract] OR "light gradient boosting machine*"[Title/Abstract] OR CatBoost[Title/Abstract] OR AdaBoost[Title/Abstract] OR "adaptive boosting"[Title/Abstract] OR bagging[Title/Abstract] OR stacking[Title/Abstract] OR "stacked generalization"[Title/Abstract] OR "stacked generalisation"[Title/Abstract] OR "ensemble learning"[Title/Abstract] OR "ensemble model*"[Title/Abstract] OR "ensemble method*"[Title/Abstract] OR "ensemble classifier*"[Title/Abstract] OR "support vector machine*"[Title/Abstract] OR "support-vector machine*"[Title/Abstract] OR "support vector classifier*"[Title/Abstract] OR SVM[Title/Abstract] OR SVC[Title/Abstract] OR "k-nearest neighbor*"[Title/Abstract] OR "k nearest neighbor*"[Title/Abstract] OR "k-nearest neighbour*"[Title/Abstract] OR "k nearest neighbour*"[Title/Abstract] OR KNN[Title/Abstract] OR "nearest neighbor algorithm*"[Title/Abstract] OR "nearest neighbour algorithm*"[Title/Abstract] OR "naive Bayes"[Title/Abstract] OR "naïve Bayes"[Title/Abstract] OR "naive Bayesian"[Title/Abstract] OR "naïve Bayesian"[Title/Abstract] OR "Bayesian classifier*"[Title/Abstract] OR "Bayesian network*"[Title/Abstract] OR "Bayesian machine learning"[Title/Abstract] OR "Bayesian additive regression tree*"[Title/Abstract] OR BART[Title/Abstract] OR "Gaussian naive Bayes"[Title/Abstract] OR "Gaussian naïve Bayes"[Title/Abstract] OR GNB[Title/Abstract] OR "fuzzy logic"[Title/Abstract] OR "fuzzy system*"[Title/Abstract] OR "fuzzy inference"[Title/Abstract] OR "genetic algorithm*"[Title/Abstract] OR "evolutionary algorithm*"[Title/Abstract] OR "genetic programming"[Title/Abstract] OR "particle swarm optimization"[Title/Abstract] OR "particle swarm optimisation"[Title/Abstract] OR "synthetic minority oversampling technique"[Title/Abstract] OR "synthetic minority over-sampling technique"[Title/Abstract] OR SMOTE[Title/Abstract] OR BSMOTE[Title/Abstract] OR "borderline SMOTE"[Title/Abstract] OR "borderline synthetic minority oversampling"[Title/Abstract] OR "minority oversampling"[Title/Abstract] OR "minority over-sampling"[Title/Abstract] OR "class imbalance"[Title/Abstract] OR "imbalanced data"[Title/Abstract]) AND ("Anesthesia, General"[MeSH Terms] OR "Anesthesiology"[MeSH Terms] OR "Perioperative Care"[MeSH Terms] OR "Perioperative Period"[MeSH Terms] OR "Preoperative Care"[MeSH Terms] OR "Intraoperative Care"[MeSH Terms] OR "Postoperative Care"[MeSH Terms] OR "Postoperative Period"[MeSH Terms] OR "Anesthesia Recovery Period"[MeSH Terms] OR "Surgical Procedures, Operative"[MeSH Terms] OR "Elective Surgical Procedures"[MeSH Terms] OR "Ambulatory Surgical Procedures"[MeSH Terms] OR "Operating Rooms"[MeSH Terms] OR "general anesthesia"[Title/Abstract] OR "general anaesthesia"[Title/Abstract] OR "general anesthetic"[Title/Abstract] OR "general anaesthetic"[Title/Abstract] OR anesthesiolog*[Title/Abstract] OR anaesthesiolog*[Title/Abstract] OR perioperativ*[Title/Abstract] OR peri-operativ*[Title/Abstract] OR perianesthe*[Title/Abstract] OR peri-anesthe*[Title/Abstract] OR perianaesthe*[Title/Abstract] OR peri-anaesthe*[Title/Abstract] OR preoperativ*[Title/Abstract] OR pre-operativ*[Title/Abstract] OR intraoperativ*[Title/Abstract] OR intra-operativ*[Title/Abstract] OR postoperativ*[Title/Abstract] OR post-operativ*[Title/Abstract] OR postanesthe*[Title/Abstract] OR post-anesthe*[Title/Abstract] OR postanaesthe*[Title/Abstract] OR post-anaesthe*[Title/Abstract] OR "post anesthesia care unit"[Title/Abstract] OR "post anaesthesia care unit"[Title/Abstract] OR "post-anesthesia care unit"[Title/Abstract] OR "post-anaesthesia care unit"[Title/Abstract] OR "postanesthesia care unit"[Title/Abstract] OR "postanaesthesia care unit"[Title/Abstract] OR PACU[Title/Abstract] OR "anesthesia recovery"[Title/Abstract] OR "anaesthesia recovery"[Title/Abstract] OR "recovery from anesthesia"[Title/Abstract] OR "recovery from anaesthesia"[Title/Abstract] OR "recovery room"[Title/Abstract] OR "operating room"[Title/Abstract] OR "operating rooms"[Title/Abstract] OR "operating theatre"[Title/Abstract] OR "operating theatres"[Title/Abstract] OR "operating theater"[Title/Abstract] OR "operating theaters"[Title/Abstract] OR surger*[Title/Abstract] OR surgical[Title/Abstract] OR operativ*[Title/Abstract] OR "surgical patient*"[Title/Abstract] OR "operative patient*"[Title/Abstract] OR "surgical procedure*"[Title/Abstract] OR "operative procedure*"[Title/Abstract] OR "ambulatory surgery"[Title/Abstract] OR "ambulatory surgical"[Title/Abstract] OR "outpatient surgery"[Title/Abstract] OR "day surgery"[Title/Abstract] OR "day-case surgery"[Title/Abstract] OR "day case surgery"[Title/Abstract] OR "inpatient surgery"[Title/Abstract] OR "major surgery"[Title/Abstract] OR "elective surgery"[Title/Abstract] OR "emergency surgery"[Title/Abstract] OR "noncardiac surgery"[Title/Abstract] OR "non-cardiac surgery"[Title/Abstract] OR "cardiac surgery"[Title/Abstract]) AND (("Postoperative Nausea and Vomiting"[MeSH Terms] OR "postoperative nausea and vomiting"[Title/Abstract] OR "post-operative nausea and vomiting"[Title/Abstract] OR PONV[Title/Abstract] OR "postoperative nausea"[Title/Abstract] OR "post-operative nausea"[Title/Abstract] OR "postoperative vomiting"[Title/Abstract] OR "post-operative vomiting"[Title/Abstract] OR "postoperative emesis"[Title/Abstract] OR "post-operative emesis"[Title/Abstract] OR "postanesthesia nausea"[Title/Abstract] OR "postanaesthesia nausea"[Title/Abstract] OR "post-anesthesia nausea"[Title/Abstract] OR "post-anaesthesia nausea"[Title/Abstract] OR "postanesthetic nausea"[Title/Abstract] OR "postanaesthetic nausea"[Title/Abstract] OR "postanesthesia vomiting"[Title/Abstract] OR "postanaesthesia vomiting"[Title/Abstract] OR "post-anesthesia vomiting"[Title/Abstract] OR "post-anaesthesia vomiting"[Title/Abstract] OR "postanesthetic vomiting"[Title/Abstract] OR "postanaesthetic vomiting"[Title/Abstract] OR ((nausea[Title/Abstract] OR vomiting[Title/Abstract] OR emesis[Title/Abstract] OR retching[Title/Abstract]) AND (postoperativ*[Title/Abstract] OR post-operativ*[Title/Abstract] OR postanesthe*[Title/Abstract] OR post-anesthe*[Title/Abstract] OR postanaesthe*[Title/Abstract] OR post-anaesthe*[Title/Abstract]))) OR ("Hypotension"[MeSH Terms] OR hypotens*[Title/Abstract] OR "intraoperative hypotension"[Title/Abstract] OR "intra-operative hypotension"[Title/Abstract] OR "perioperative hypotension"[Title/Abstract] OR "peri-operative hypotension"[Title/Abstract] OR "postoperative hypotension"[Title/Abstract] OR "post-operative hypotension"[Title/Abstract] OR "postanesthesia care unit hypotension"[Title/Abstract] OR "postanaesthesia care unit hypotension"[Title/Abstract] OR "post-anesthesia care unit hypotension"[Title/Abstract] OR "post-anaesthesia care unit hypotension"[Title/Abstract] OR "PACU hypotension"[Title/Abstract] OR "postinduction hypotension"[Title/Abstract] OR "post-induction hypotension"[Title/Abstract] OR "hypotension after induction"[Title/Abstract] OR "hypotension following induction"[Title/Abstract] OR "anesthesia-induced hypotension"[Title/Abstract] OR "anaesthesia-induced hypotension"[Title/Abstract] OR "anesthesia related hypotension"[Title/Abstract] OR "anaesthesia related hypotension"[Title/Abstract] OR "arterial hypotension"[Title/Abstract] OR "low blood pressure"[Title/Abstract] OR "low arterial pressure"[Title/Abstract] OR "low mean arterial pressure"[Title/Abstract] OR "mean arterial pressure"[Title/Abstract] OR "hemodynamic instability"[Title/Abstract] OR "haemodynamic instability"[Title/Abstract] OR "circulatory instability"[Title/Abstract] OR "blood pressure instability"[Title/Abstract] OR "hypotension prediction index"[Title/Abstract] OR vasopleg*[Title/Abstract]) OR (("Intensive Care Units"[MeSH Terms] OR "Critical Care"[MeSH Terms] OR ICU[Title/Abstract] OR ICUs[Title/Abstract] OR "intensive care"[Title/Abstract] OR "intensive care unit"[Title/Abstract] OR "intensive care units"[Title/Abstract] OR "critical care"[Title/Abstract] OR "critical care unit"[Title/Abstract] OR "critical care units"[Title/Abstract]) AND ("Length of Stay"[MeSH Terms] OR "length of stay"[Title/Abstract] OR "ICU length of stay"[Title/Abstract] OR "intensive care length of stay"[Title/Abstract] OR "critical care length of stay"[Title/Abstract] OR "ICU stay"[Title/Abstract] OR "intensive care stay"[Title/Abstract] OR "critical care stay"[Title/Abstract] OR "prolonged stay"[Title/Abstract] OR "extended stay"[Title/Abstract] OR "long stay"[Title/Abstract] OR "prolonged ICU"[Title/Abstract] OR "extended ICU"[Title/Abstract] OR "long ICU"[Title/Abstract] OR "prolonged intensive care"[Title/Abstract] OR "extended intensive care"[Title/Abstract] OR "ICU admission"[Title/Abstract] OR "ICU requirement"[Title/Abstract] OR "intensive care admission"[Title/Abstract] OR "critical care admission"[Title/Abstract] OR "need for intensive care"[Title/Abstract] OR "requirement for intensive care"[Title/Abstract] OR "unplanned ICU admission"[Title/Abstract] OR "unplanned intensive care admission"[Title/Abstract] OR "postoperative ICU admission"[Title/Abstract] OR "post-operative ICU admission"[Title/Abstract])) OR ("Mortality"[MeSH Terms] OR "Hospital Mortality"[MeSH Terms] OR "Fatal Outcome"[MeSH Terms] OR mortality[Title/Abstract] OR death[Title/Abstract] OR deaths[Title/Abstract] OR fatal*[Title/Abstract] OR "in-hospital mortality"[Title/Abstract] OR "in hospital mortality"[Title/Abstract] OR "hospital mortality"[Title/Abstract] OR "inpatient mortality"[Title/Abstract] OR "perioperative mortality"[Title/Abstract] OR "peri-operative mortality"[Title/Abstract] OR "postoperative mortality"[Title/Abstract] OR "post-operative mortality"[Title/Abstract] OR "operative mortality"[Title/Abstract] OR "surgical mortality"[Title/Abstract] OR "in-hospital death"[Title/Abstract] OR "in hospital death"[Title/Abstract] OR "hospital death"[Title/Abstract] OR "postoperative death"[Title/Abstract] OR "post-operative death"[Title/Abstract] OR "perioperative death"[Title/Abstract] OR "peri-operative death"[Title/Abstract] OR "operative death"[Title/Abstract] OR "death after surgery"[Title/Abstract] OR "death following surgery"[Title/Abstract] OR "30-day mortality"[Title/Abstract] OR "thirty-day mortality"[Title/Abstract] OR "short-term mortality"[Title/Abstract] OR "all-cause mortality"[Title/Abstract]) OR ("Neurologic Manifestations"[MeSH Terms] OR "Nervous System Diseases"[MeSH Terms] OR "Postoperative Cognitive Complications"[MeSH Terms] OR "Cognitive Dysfunction"[MeSH Terms] OR "Delirium"[MeSH Terms] OR "Emergence Delirium"[MeSH Terms] OR "Stroke"[MeSH Terms] OR "Cerebrovascular Disorders"[MeSH Terms] OR "Brain Ischemia"[MeSH Terms] OR "Cerebral Infarction"[MeSH Terms] OR "Seizures"[MeSH Terms] OR "Coma"[MeSH Terms] OR "neurological complication*"[Title/Abstract] OR "neurologic complication*"[Title/Abstract] OR "neurological outcome*"[Title/Abstract] OR "neurologic outcome*"[Title/Abstract] OR "neurological morbidity"[Title/Abstract] OR "neurologic morbidity"[Title/Abstract] OR "postoperative neurological complication*"[Title/Abstract] OR "postoperative neurologic complication*"[Title/Abstract] OR "post-operative neurological complication*"[Title/Abstract] OR "post-operative neurologic complication*"[Title/Abstract] OR "perioperative neurological complication*"[Title/Abstract] OR "perioperative neurologic complication*"[Title/Abstract] OR "central nervous system complication*"[Title/Abstract] OR "CNS complication*"[Title/Abstract] OR "perioperative neurocognitive disorder*"[Title/Abstract] OR "peri-operative neurocognitive disorder*"[Title/Abstract] OR "postoperative neurocognitive disorder*"[Title/Abstract] OR "post-operative neurocognitive disorder*"[Title/Abstract] OR "postoperative cognitive dysfunction"[Title/Abstract] OR "post-operative cognitive dysfunction"[Title/Abstract] OR POCD[Title/Abstract] OR "postoperative cognitive decline"[Title/Abstract] OR "post-operative cognitive decline"[Title/Abstract] OR "cognitive decline"[Title/Abstract] OR "cognitive impairment"[Title/Abstract] OR "postoperative delirium"[Title/Abstract] OR "post-operative delirium"[Title/Abstract] OR "emergence delirium"[Title/Abstract] OR "perioperative delirium"[Title/Abstract] OR "peri-operative delirium"[Title/Abstract] OR "postoperative stroke"[Title/Abstract] OR "post-operative stroke"[Title/Abstract] OR "perioperative stroke"[Title/Abstract] OR "peri-operative stroke"[Title/Abstract] OR "cerebrovascular accident*"[Title/Abstract] OR "cerebral ischemia"[Title/Abstract] OR "cerebral ischaemia"[Title/Abstract] OR "brain ischemia"[Title/Abstract] OR "brain ischaemia"[Title/Abstract] OR "cerebral infarction"[Title/Abstract] OR "brain infarction"[Title/Abstract] OR "brain injury"[Title/Abstract] OR encephalopath*[Title/Abstract] OR seizure*[Title/Abstract] OR coma[Title/Abstract] OR "neurological deficit*"[Title/Abstract] OR "neurologic deficit*"[Title/Abstract])) AND ("Risk Assessment"[MeSH Terms] OR "Prognosis"[MeSH Terms] OR "Predictive Value of Tests"[MeSH Terms] OR "Models, Statistical"[MeSH Terms] OR "ROC Curve"[MeSH Terms] OR "Sensitivity and Specificity"[MeSH Terms] OR "Nomograms"[MeSH Terms] OR predict*[Title/Abstract] OR prognos*[Title/Abstract] OR forecast*[Title/Abstract] OR "risk prediction"[Title/Abstract] OR "risk-prediction"[Title/Abstract] OR "risk model*"[Title/Abstract] OR "prediction model*"[Title/Abstract] OR "predictive model*"[Title/Abstract] OR "prognostic model*"[Title/Abstract] OR "risk score*"[Title/Abstract] OR "prediction score*"[Title/Abstract] OR "risk stratification"[Title/Abstract] OR "risk assessment"[Title/Abstract] OR "clinical prediction"[Title/Abstract] OR "prediction tool*"[Title/Abstract] OR "predictive tool*"[Title/Abstract] OR "prediction rule*"[Title/Abstract] OR "clinical prediction rule*"[Title/Abstract] OR "clinical decision rule*"[Title/Abstract] OR classifier*[Title/Abstract] OR classification[Title/Abstract] OR discriminat*[Title/Abstract] OR calibrat*[Title/Abstract] OR validat*[Title/Abstract] OR "internal validation"[Title/Abstract] OR "external validation"[Title/Abstract] OR "temporal validation"[Title/Abstract] OR "geographical validation"[Title/Abstract] OR "geographic validation"[Title/Abstract] OR "cross-validation"[Title/Abstract] OR "cross validation"[Title/Abstract] OR "model development"[Title/Abstract] OR "model derivation"[Title/Abstract] OR "model evaluation"[Title/Abstract] OR "model performance"[Title/Abstract] OR "predictive performance"[Title/Abstract] OR "prediction performance"[Title/Abstract] OR accuracy[Title/Abstract] OR sensitivity[Title/Abstract] OR specificity[Title/Abstract] OR AUROC[Title/Abstract] OR AUC[Title/Abstract] OR "AUC-ROC"[Title/Abstract] OR "area under the curve"[Title/Abstract] OR "area under the receiver operating characteristic curve"[Title/Abstract] OR "receiver operating characteristic"[Title/Abstract] OR "ROC curve"[Title/Abstract] OR "c-statistic"[Title/Abstract] OR "c statistic"[Title/Abstract] OR "concordance statistic"[Title/Abstract] OR "Brier score"[Title/Abstract] OR "decision curve"[Title/Abstract] OR "decision curve analysis"[Title/Abstract] OR "early warning"[Title/Abstract] OR "probability estimation"[Title/Abstract] OR "individualized risk"[Title/Abstract] OR "individualised risk"[Title/Abstract])
Embase	('artificial intelligence'/exp OR 'machine learning'/exp OR 'deep learning'/exp OR 'neural network'/exp OR 'data mining'/exp OR 'decision tree'/exp OR 'random forest'/exp OR 'support vector machine'/exp OR 'pattern recognition'/exp OR 'expert system'/exp OR 'fuzzy logic'/exp OR 'genetic algorithm'/exp OR 'algorithm'/exp OR 'artificial intelligence':ti,ab,kw OR 'artificial-intelligence':ti,ab,kw OR AI:ti,ab,kw OR 'machine intelligence':ti,ab,kw OR 'computational intelligence':ti,ab,kw OR 'machine learning':ti,ab,kw OR 'machine-learning':ti,ab,kw OR ML:ti,ab,kw OR 'deep learning':ti,ab,kw OR 'deep-learning':ti,ab,kw OR 'representation learning':ti,ab,kw OR 'supervised learning':ti,ab,kw OR 'unsupervised learning':ti,ab,kw OR 'semi-supervised learning':ti,ab,kw OR 'reinforcement learning':ti,ab,kw OR 'transfer learning':ti,ab,kw OR 'federated learning':ti,ab,kw OR 'statistical learning':ti,ab,kw OR 'automated learning':ti,ab,kw OR 'predictive analytics':ti,ab,kw OR 'data mining':ti,ab,kw OR 'pattern recognition':ti,ab,kw OR 'pattern analysis':ti,ab,kw OR 'expert system*':ti,ab,kw OR 'knowledge-based system*':ti,ab,kw OR 'intelligent system*':ti,ab,kw OR 'clinical decision support system*':ti,ab,kw OR 'artificial neural network*':ti,ab,kw OR 'neural network*':ti,ab,kw OR ANN:ti,ab,kw OR DNN:ti,ab,kw OR CNN:ti,ab,kw OR RNN:ti,ab,kw OR LSTM:ti,ab,kw OR GRU:ti,ab,kw OR 'deep neural network*':ti,ab,kw OR 'convolutional neural network*':ti,ab,kw OR 'recurrent neural network*':ti,ab,kw OR 'long short-term memory':ti,ab,kw OR 'long short term memory':ti,ab,kw OR 'gated recurrent unit*':ti,ab,kw OR 'multilayer perceptron*':ti,ab,kw OR 'multi-layer perceptron*':ti,ab,kw OR MLP:ti,ab,kw OR 'feedforward neural network*':ti,ab,kw OR 'feed-forward neural network*':ti,ab,kw OR 'backpropagation neural network*':ti,ab,kw OR 'back-propagation neural network*':ti,ab,kw OR 'probabilistic neural network*':ti,ab,kw OR 'self-organizing map*':ti,ab,kw OR 'self organising map*':ti,ab,kw OR 'decision tree*':ti,ab,kw OR 'classification tree*':ti,ab,kw OR 'regression tree*':ti,ab,kw OR CART:ti,ab,kw OR 'C4.5':ti,ab,kw OR 'C5.0':ti,ab,kw OR 'random forest*':ti,ab,kw OR 'balanced random forest*':ti,ab,kw OR 'conditional random forest*':ti,ab,kw OR 'conditional inference forest*':ti,ab,kw OR cforest:ti,ab,kw OR 'extremely randomized tree*':ti,ab,kw OR 'extremely randomised tree*':ti,ab,kw OR 'extra tree*':ti,ab,kw OR 'gradient boosting':ti,ab,kw OR 'gradient-boosting':ti,ab,kw OR 'gradient boosting machine*':ti,ab,kw OR GBM:ti,ab,kw OR GBDT:ti,ab,kw OR 'gradient boosting decision tree*':ti,ab,kw OR 'extreme gradient boosting':ti,ab,kw OR XGBoost:ti,ab,kw OR LightGBM:ti,ab,kw OR 'light gradient boosting machine*':ti,ab,kw OR CatBoost:ti,ab,kw OR AdaBoost:ti,ab,kw OR 'adaptive boosting':ti,ab,kw OR bagging:ti,ab,kw OR stacking:ti,ab,kw OR 'stacked generalization':ti,ab,kw OR 'stacked generalisation':ti,ab,kw OR 'ensemble learning':ti,ab,kw OR 'ensemble model*':ti,ab,kw OR 'ensemble method*':ti,ab,kw OR 'ensemble classifier*':ti,ab,kw OR 'support vector machine*':ti,ab,kw OR 'support-vector machine*':ti,ab,kw OR 'support vector classifier*':ti,ab,kw OR SVM:ti,ab,kw OR SVC:ti,ab,kw OR 'k-nearest neighbor*':ti,ab,kw OR 'k nearest neighbor*':ti,ab,kw OR 'k-nearest neighbour*':ti,ab,kw OR 'k nearest neighbour*':ti,ab,kw OR KNN:ti,ab,kw OR 'nearest neighbor algorithm*':ti,ab,kw OR 'nearest neighbour algorithm*':ti,ab,kw OR 'naive Bayes':ti,ab,kw OR 'naïve Bayes':ti,ab,kw OR 'naive Bayesian':ti,ab,kw OR 'naïve Bayesian':ti,ab,kw OR 'Bayesian classifier*':ti,ab,kw OR 'Bayesian network*':ti,ab,kw OR 'Bayesian machine learning':ti,ab,kw OR 'Bayesian additive regression tree*':ti,ab,kw OR BART:ti,ab,kw OR 'Gaussian naive Bayes':ti,ab,kw OR 'Gaussian naïve Bayes':ti,ab,kw OR GNB:ti,ab,kw OR 'linear discriminant analysis':ti,ab,kw OR LDA:ti,ab,kw OR 'quadratic discriminant analysis':ti,ab,kw OR 'logistic model tree*':ti,ab,kw OR 'fuzzy logic':ti,ab,kw OR 'fuzzy system*':ti,ab,kw OR 'fuzzy inference':ti,ab,kw OR 'genetic algorithm*':ti,ab,kw OR 'evolutionary algorithm*':ti,ab,kw OR 'genetic programming':ti,ab,kw OR 'particle swarm optimization':ti,ab,kw OR 'particle swarm optimisation':ti,ab,kw OR 'synthetic minority oversampling technique':ti,ab,kw OR 'synthetic minority over-sampling technique':ti,ab,kw OR SMOTE:ti,ab,kw OR BSMOTE:ti,ab,kw OR 'borderline SMOTE':ti,ab,kw OR 'borderline synthetic minority oversampling':ti,ab,kw OR 'minority oversampling':ti,ab,kw OR 'minority over-sampling':ti,ab,kw OR 'class imbalance':ti,ab,kw OR 'imbalanced data':ti,ab,kw) AND ('general anesthesia'/exp OR 'anesthesiology'/exp OR 'perioperative period'/exp OR 'preoperative care'/exp OR 'intraoperative care'/exp OR 'postoperative care'/exp OR 'postoperative period'/exp OR 'postanesthesia care unit'/exp OR 'anesthesia recovery'/exp OR 'surgery'/exp OR 'surgical procedure'/exp OR 'elective surgery'/exp OR 'ambulatory surgery'/exp OR 'operating room'/exp OR 'general anesthesia':ti,ab,kw OR 'general anaesthesia':ti,ab,kw OR 'general anesthetic':ti,ab,kw OR 'general anaesthetic':ti,ab,kw OR anesthesiolog*:ti,ab,kw OR anaesthesiolog*:ti,ab,kw OR perioperativ*:ti,ab,kw OR peri-operativ*:ti,ab,kw OR perianesthe*:ti,ab,kw OR peri-anesthe*:ti,ab,kw OR perianaesthe*:ti,ab,kw OR peri-anaesthe*:ti,ab,kw OR preoperativ*:ti,ab,kw OR pre-operativ*:ti,ab,kw OR intraoperativ*:ti,ab,kw OR intra-operativ*:ti,ab,kw OR postoperativ*:ti,ab,kw OR post-operativ*:ti,ab,kw OR postanesthe*:ti,ab,kw OR post-anesthe*:ti,ab,kw OR postanaesthe*:ti,ab,kw OR post-anaesthe*:ti,ab,kw OR 'post anesthesia care unit':ti,ab,kw OR 'post anaesthesia care unit':ti,ab,kw OR 'post-anesthesia care unit':ti,ab,kw OR 'post-anaesthesia care unit':ti,ab,kw OR 'postanesthesia care unit':ti,ab,kw OR 'postanaesthesia care unit':ti,ab,kw OR PACU:ti,ab,kw OR 'anesthesia recovery':ti,ab,kw OR 'anaesthesia recovery':ti,ab,kw OR 'recovery from anesthesia':ti,ab,kw OR 'recovery from anaesthesia':ti,ab,kw OR 'recovery room':ti,ab,kw OR 'operating room':ti,ab,kw OR 'operating rooms':ti,ab,kw OR 'operating theatre':ti,ab,kw OR 'operating theatres':ti,ab,kw OR 'operating theater':ti,ab,kw OR 'operating theaters':ti,ab,kw OR surger*:ti,ab,kw OR surgical:ti,ab,kw OR operativ*:ti,ab,kw OR 'surgical patient*':ti,ab,kw OR 'operative patient*':ti,ab,kw OR 'surgical procedure*':ti,ab,kw OR 'operative procedure*':ti,ab,kw OR 'ambulatory surgery':ti,ab,kw OR 'ambulatory surgical':ti,ab,kw OR 'outpatient surgery':ti,ab,kw OR 'day surgery':ti,ab,kw OR 'day-case surgery':ti,ab,kw OR 'day case surgery':ti,ab,kw OR 'inpatient surgery':ti,ab,kw OR 'major surgery':ti,ab,kw OR 'elective surgery':ti,ab,kw OR 'emergency surgery':ti,ab,kw OR 'noncardiac surgery':ti,ab,kw OR 'non-cardiac surgery':ti,ab,kw OR 'cardiac surgery':ti,ab,kw) AND (('postoperative nausea and vomiting'/exp OR 'postoperative nausea and vomiting':ti,ab,kw OR 'post-operative nausea and vomiting':ti,ab,kw OR PONV:ti,ab,kw OR 'postoperative nausea':ti,ab,kw OR 'post-operative nausea':ti,ab,kw OR 'postoperative vomiting':ti,ab,kw OR 'post-operative vomiting':ti,ab,kw OR 'postoperative emesis':ti,ab,kw OR 'post-operative emesis':ti,ab,kw OR 'postanesthesia nausea':ti,ab,kw OR 'postanaesthesia nausea':ti,ab,kw OR 'post-anesthesia nausea':ti,ab,kw OR 'post-anaesthesia nausea':ti,ab,kw OR 'postanesthetic nausea':ti,ab,kw OR 'postanaesthetic nausea':ti,ab,kw OR 'postanesthesia vomiting':ti,ab,kw OR 'postanaesthesia vomiting':ti,ab,kw OR 'post-anesthesia vomiting':ti,ab,kw OR 'post-anaesthesia vomiting':ti,ab,kw OR 'postanesthetic vomiting':ti,ab,kw OR 'postanaesthetic vomiting':ti,ab,kw OR ((postoperativ*:ti,ab,kw OR post-operativ*:ti,ab,kw OR postanesthe*:ti,ab,kw OR post-anesthe*:ti,ab,kw OR postanaesthe*:ti,ab,kw OR post-anaesthe*:ti,ab,kw) NEAR/3 (nausea:ti,ab,kw OR vomit*:ti,ab,kw OR emesis:ti,ab,kw OR retching:ti,ab,kw))) OR ('hypotension'/exp OR hypotens*:ti,ab,kw OR 'intraoperative hypotension':ti,ab,kw OR 'intra-operative hypotension':ti,ab,kw OR 'perioperative hypotension':ti,ab,kw OR 'peri-operative hypotension':ti,ab,kw OR 'postoperative hypotension':ti,ab,kw OR 'post-operative hypotension':ti,ab,kw OR 'postanesthesia care unit hypotension':ti,ab,kw OR 'postanaesthesia care unit hypotension':ti,ab,kw OR 'post-anesthesia care unit hypotension':ti,ab,kw OR 'post-anaesthesia care unit hypotension':ti,ab,kw OR 'PACU hypotension':ti,ab,kw OR 'postinduction hypotension':ti,ab,kw OR 'post-induction hypotension':ti,ab,kw OR 'hypotension after induction':ti,ab,kw OR 'hypotension following induction':ti,ab,kw OR 'anesthesia-induced hypotension':ti,ab,kw OR 'anaesthesia-induced hypotension':ti,ab,kw OR 'anesthesia related hypotension':ti,ab,kw OR 'anaesthesia related hypotension':ti,ab,kw OR 'arterial hypotension':ti,ab,kw OR 'low blood pressure':ti,ab,kw OR 'low arterial pressure':ti,ab,kw OR 'low mean arterial pressure':ti,ab,kw OR 'mean arterial pressure':ti,ab,kw OR 'hemodynamic instability':ti,ab,kw OR 'haemodynamic instability':ti,ab,kw OR 'circulatory instability':ti,ab,kw OR 'blood pressure instability':ti,ab,kw OR 'hypotension prediction index':ti,ab,kw OR vasopleg*:ti,ab,kw) OR (('intensive care unit'/exp OR 'critical care'/exp OR ICU:ti,ab,kw OR ICUs:ti,ab,kw OR 'intensive care':ti,ab,kw OR 'intensive care unit':ti,ab,kw OR 'intensive care units':ti,ab,kw OR 'critical care':ti,ab,kw OR 'critical care unit':ti,ab,kw OR 'critical care units':ti,ab,kw) AND ('length of stay'/exp OR 'length of stay':ti,ab,kw OR 'ICU length of stay':ti,ab,kw OR 'intensive care length of stay':ti,ab,kw OR 'critical care length of stay':ti,ab,kw OR 'ICU stay':ti,ab,kw OR 'intensive care stay':ti,ab,kw OR 'critical care stay':ti,ab,kw OR 'prolonged stay':ti,ab,kw OR 'extended stay':ti,ab,kw OR 'long stay':ti,ab,kw OR 'prolonged ICU':ti,ab,kw OR 'extended ICU':ti,ab,kw OR 'long ICU':ti,ab,kw OR 'prolonged intensive care':ti,ab,kw OR 'extended intensive care':ti,ab,kw OR 'ICU admission':ti,ab,kw OR 'ICU requirement':ti,ab,kw OR 'intensive care admission':ti,ab,kw OR 'critical care admission':ti,ab,kw OR 'need for intensive care':ti,ab,kw OR 'requirement for intensive care':ti,ab,kw OR 'unplanned ICU admission':ti,ab,kw OR 'unplanned intensive care admission':ti,ab,kw OR 'postoperative ICU admission':ti,ab,kw OR 'post-operative ICU admission':ti,ab,kw)) OR ('mortality'/exp OR 'hospital mortality'/exp OR 'death'/exp OR mortality:ti,ab,kw OR death:ti,ab,kw OR deaths:ti,ab,kw OR fatal*:ti,ab,kw OR 'in-hospital mortality':ti,ab,kw OR 'in hospital mortality':ti,ab,kw OR 'hospital mortality':ti,ab,kw OR 'inpatient mortality':ti,ab,kw OR 'perioperative mortality':ti,ab,kw OR 'peri-operative mortality':ti,ab,kw OR 'postoperative mortality':ti,ab,kw OR 'post-operative mortality':ti,ab,kw OR 'operative mortality':ti,ab,kw OR 'surgical mortality':ti,ab,kw OR 'in-hospital death':ti,ab,kw OR 'in hospital death':ti,ab,kw OR 'hospital death':ti,ab,kw OR 'postoperative death':ti,ab,kw OR 'post-operative death':ti,ab,kw OR 'perioperative death':ti,ab,kw OR 'peri-operative death':ti,ab,kw OR 'operative death':ti,ab,kw OR 'death after surgery':ti,ab,kw OR 'death following surgery':ti,ab,kw OR '30-day mortality':ti,ab,kw OR 'thirty-day mortality':ti,ab,kw OR 'short-term mortality':ti,ab,kw OR 'all-cause mortality':ti,ab,kw) OR ('neurologic disease'/exp OR 'postoperative complication'/exp OR 'delirium'/exp OR 'postoperative delirium'/exp OR 'cognitive defect'/exp OR 'stroke'/exp OR 'brain ischemia'/exp OR 'cerebral infarction'/exp OR 'epileptic seizure'/exp OR 'coma'/exp OR 'neurological complication*':ti,ab,kw OR 'neurologic complication*':ti,ab,kw OR 'neurological outcome*':ti,ab,kw OR 'neurologic outcome*':ti,ab,kw OR 'neurological morbidity':ti,ab,kw OR 'neurologic morbidity':ti,ab,kw OR 'postoperative neurological complication*':ti,ab,kw OR 'postoperative neurologic complication*':ti,ab,kw OR 'post-operative neurological complication*':ti,ab,kw OR 'post-operative neurologic complication*':ti,ab,kw OR 'perioperative neurological complication*':ti,ab,kw OR 'perioperative neurologic complication*':ti,ab,kw OR 'central nervous system complication*':ti,ab,kw OR 'CNS complication*':ti,ab,kw OR 'perioperative neurocognitive disorder*':ti,ab,kw OR 'peri-operative neurocognitive disorder*':ti,ab,kw OR 'postoperative neurocognitive disorder*':ti,ab,kw OR 'post-operative neurocognitive disorder*':ti,ab,kw OR 'postoperative cognitive dysfunction':ti,ab,kw OR 'post-operative cognitive dysfunction':ti,ab,kw OR POCD:ti,ab,kw OR 'postoperative cognitive decline':ti,ab,kw OR 'post-operative cognitive decline':ti,ab,kw OR 'cognitive decline':ti,ab,kw OR 'cognitive impairment':ti,ab,kw OR 'postoperative delirium':ti,ab,kw OR 'post-operative delirium':ti,ab,kw OR 'emergence delirium':ti,ab,kw OR 'perioperative delirium':ti,ab,kw OR 'peri-operative delirium':ti,ab,kw OR 'postoperative stroke':ti,ab,kw OR 'post-operative stroke':ti,ab,kw OR 'perioperative stroke':ti,ab,kw OR 'peri-operative stroke':ti,ab,kw OR 'cerebrovascular accident*':ti,ab,kw OR 'cerebral ischemia':ti,ab,kw OR 'cerebral ischaemia':ti,ab,kw OR 'brain ischemia':ti,ab,kw OR 'brain ischaemia':ti,ab,kw OR 'cerebral infarction':ti,ab,kw OR 'brain infarction':ti,ab,kw OR 'brain injury':ti,ab,kw OR encephalopath*:ti,ab,kw OR seizure*:ti,ab,kw OR coma:ti,ab,kw OR 'neurological deficit*':ti,ab,kw OR 'neurologic deficit*':ti,ab,kw)) AND ('prediction'/exp OR 'risk assessment'/exp OR 'prognosis'/exp OR 'predictive value'/exp OR 'statistical model'/exp OR 'receiver operating characteristic'/exp OR 'sensitivity and specificity'/exp OR 'nomogram'/exp OR 'model validation'/exp OR predict*:ti,ab,kw OR prognos*:ti,ab,kw OR forecast*:ti,ab,kw OR 'risk prediction':ti,ab,kw OR 'risk-prediction':ti,ab,kw OR 'risk model*':ti,ab,kw OR 'prediction model*':ti,ab,kw OR 'predictive model*':ti,ab,kw OR 'prognostic model*':ti,ab,kw OR 'risk score*':ti,ab,kw OR 'prediction score*':ti,ab,kw OR 'risk stratification':ti,ab,kw OR 'risk assessment':ti,ab,kw OR 'clinical prediction':ti,ab,kw OR 'prediction tool*':ti,ab,kw OR 'predictive tool*':ti,ab,kw OR 'prediction rule*':ti,ab,kw OR 'clinical prediction rule*':ti,ab,kw OR 'clinical decision rule*':ti,ab,kw OR classifier*:ti,ab,kw OR classification:ti,ab,kw OR discriminat*:ti,ab,kw OR calibrat*:ti,ab,kw OR validat*:ti,ab,kw OR 'internal validation':ti,ab,kw OR 'external validation':ti,ab,kw OR 'temporal validation':ti,ab,kw OR 'geographical validation':ti,ab,kw OR 'geographic validation':ti,ab,kw OR 'cross-validation':ti,ab,kw OR 'cross validation':ti,ab,kw OR 'model development':ti,ab,kw OR 'model derivation':ti,ab,kw OR 'model evaluation':ti,ab,kw OR 'model performance':ti,ab,kw OR 'predictive performance':ti,ab,kw OR 'prediction performance':ti,ab,kw OR accuracy:ti,ab,kw OR sensitivity:ti,ab,kw OR specificity:ti,ab,kw OR AUROC:ti,ab,kw OR AUC:ti,ab,kw OR 'AUC-ROC':ti,ab,kw OR 'area under the curve':ti,ab,kw OR 'area under the receiver operating characteristic curve':ti,ab,kw OR 'receiver operating characteristic':ti,ab,kw OR 'ROC curve':ti,ab,kw OR 'c-statistic':ti,ab,kw OR 'c statistic':ti,ab,kw OR 'concordance statistic':ti,ab,kw OR 'Brier score':ti,ab,kw OR 'decision curve':ti,ab,kw OR 'decision curve analysis':ti,ab,kw OR 'early warning':ti,ab,kw OR 'probability estimation':ti,ab,kw OR 'individualized risk':ti,ab,kw OR 'individualised risk':ti,ab,kw)
Scopus	TITLE-ABS-KEY("artificial intelligence" OR "artificial-intelligence" OR AI OR "machine intelligence" OR "computational intelligence" OR "intelligent computing" OR "machine learning" OR "machine-learning" OR ML OR "deep learning" OR "deep-learning" OR "representation learning" OR "supervised learning" OR "unsupervised learning" OR "semi-supervised learning" OR "semisupervised learning" OR "reinforcement learning" OR "transfer learning" OR "federated learning" OR "statistical learning" OR "automated learning" OR "automated machine learning" OR AutoML OR "predictive analytics" OR "data mining" OR "pattern recognition" OR "pattern analysis" OR "machine pattern recognition" OR "expert system*" OR "knowledge-based system*" OR "knowledge based system*" OR "intelligent system*" OR "clinical decision support system*" OR "computer-aided decision*" OR "computer aided decision*" OR "computer-assisted decision*" OR "computer assisted decision*" OR "artificial neural network*" OR "neural network*" OR ANN OR DNN OR CNN OR RNN OR LSTM OR GRU OR "deep neural network*" OR "convolutional neural network*" OR "recurrent neural network*" OR "recursive neural network*" OR "long short-term memory" OR "long short term memory" OR "gated recurrent unit*" OR "multilayer perceptron*" OR "multi-layer perceptron*" OR MLP OR "feedforward neural network*" OR "feed-forward neural network*" OR "backpropagation neural network*" OR "back-propagation neural network*" OR "probabilistic neural network*" OR "radial basis function network*" OR "self-organizing map*" OR "self organising map*" OR "transformer model*" OR "transformer network*" OR "attention-based model*" OR "attention based model*" OR "decision tree*" OR "classification tree*" OR "regression tree*" OR CART OR "C4.5" OR "C5.0" OR "random forest*" OR "balanced random forest*" OR "conditional random forest*" OR "conditional inference forest*" OR cforest OR "extremely randomized tree*" OR "extremely randomised tree*" OR "extra tree*" OR "extra-tree*" OR "gradient boosting" OR "gradient-boosting" OR "gradient boosting machine*" OR GBM OR GBDT OR "gradient boosting decision tree*" OR "extreme gradient boosting" OR XGBoost OR LightGBM OR "light gradient boosting machine*" OR CatBoost OR AdaBoost OR "adaptive boosting" OR "boosted tree*" OR "boosting algorithm*" OR bagging OR "bootstrap aggregating" OR stacking OR "stacked generalization" OR "stacked generalisation" OR "ensemble learning" OR "ensemble model*" OR "ensemble method*" OR "ensemble classifier*" OR "ensemble algorithm*" OR "voting classifier*" OR "support vector machine*" OR "support-vector machine*" OR "support vector classifier*" OR "support-vector classifier*" OR SVM OR SVC OR "kernel machine*" OR "kernel method*" OR "k-nearest neighbor*" OR "k nearest neighbor*" OR "k-nearest neighbour*" OR "k nearest neighbour*" OR KNN OR "nearest neighbor algorithm*" OR "nearest neighbour algorithm*" OR "nearest-neighbor classifier*" OR "nearest-neighbour classifier*" OR "naive Bayes" OR "naïve Bayes" OR "naive Bayesian" OR "naïve Bayesian" OR "Bayesian classifier*" OR "Bayes classifier*" OR "Bayesian network*" OR "Bayesian machine learning" OR "Bayesian additive regression tree*" OR BART OR "Gaussian naive Bayes" OR "Gaussian naïve Bayes" OR GNB OR "Gaussian process*" OR "linear discriminant analysis" OR LDA OR "quadratic discriminant analysis" OR QDA OR "discriminant analysis classifier*" OR "logistic model tree*" OR "fuzzy logic" OR "fuzzy system*" OR "fuzzy inference" OR "neuro-fuzzy" OR "neuro fuzzy" OR "genetic algorithm*" OR "evolutionary algorithm*" OR "evolutionary computation" OR "genetic programming" OR "particle swarm optimization" OR "particle swarm optimisation" OR "ant colony optimization" OR "ant colony optimisation" OR "synthetic minority oversampling technique" OR "synthetic minority over-sampling technique" OR SMOTE OR BSMOTE OR Borderline-SMOTE OR "borderline SMOTE" OR "borderline synthetic minority oversampling" OR "minority oversampling" OR "minority over-sampling" OR "random oversampling" OR "random over-sampling" OR "class imbalance" OR "class-imbalance" OR "imbalanced data" OR "imbalanced dataset*" OR "imbalanced learning" OR "cost-sensitive learning" OR "cost sensitive learning" OR "recursive feature elimination" OR RFE OR "feature selection algorithm*" OR "learning algorithm*" OR "classification algorithm*" OR "predictive algorithm*" OR "prediction algorithm*" OR "explainable artificial intelligence" OR "explainable AI" OR XAI OR SHAP OR "Shapley additive explanation*" OR LIME) AND TITLE-ABS-KEY("general anesthesia" OR "general anaesthesia" OR "general anesthetic*" OR "general anaesthetic*" OR ((general) W/2 (anesthe* OR anaesthe*)) OR anesthesiolog* OR anaesthesiolog* OR anesthesia OR anaesthesia OR anesthetic* OR anaesthetic* OR perioperativ* OR peri-operativ* OR perianesthe* OR peri-anesthe* OR perianaesthe* OR peri-anaesthe* OR preoperativ* OR pre-operativ* OR intraoperativ* OR intra-operativ* OR postoperativ* OR post-operativ* OR postanesthe* OR post-anesthe* OR postanaesthe* OR post-anaesthe* OR "post anesthesia care unit" OR "post anaesthesia care unit" OR "post-anesthesia care unit" OR "post-anaesthesia care unit" OR "postanesthesia care unit" OR "postanaesthesia care unit" OR PACU OR "anesthesia recovery" OR "anaesthesia recovery" OR "recovery from anesthesia" OR "recovery from anaesthesia" OR "anesthetic recovery" OR "anaesthetic recovery" OR "recovery room*" OR "operating room*" OR "operating theatre*" OR "operating theater*" OR "surgical theatre*" OR "surgical theater*" OR surger* OR surgical OR operativ* OR "surgical patient*" OR "operative patient*" OR "surgical population*" OR "surgical procedure*" OR "operative procedure*" OR "surgical operation*" OR "ambulatory surgery" OR "ambulatory surgical procedure*" OR "outpatient surgery" OR "outpatient surgical procedure*" OR "day surgery" OR "day-case surgery" OR "day case surgery" OR "day-care surgery" OR "day care surgery" OR "inpatient surgery" OR "in-patient surgery" OR "major surgery" OR "major surgical procedure*" OR "elective surgery" OR "elective surgical procedure*" OR "emergency surgery" OR "emergency surgical procedure*" OR "urgent surgery" OR "noncardiac surgery" OR "non-cardiac surgery" OR "cardiac surgery" OR "cardiovascular surgery" OR "general surgery" OR "abdominal surgery" OR "gastrointestinal surgery" OR "laparoscopic surgery" OR "laparoscopic procedure*" OR "orthopedic surgery" OR "orthopaedic surgery" OR "neurosurgery" OR "neurological surgery" OR "gynecologic surgery" OR "gynaecologic surgery" OR "urologic surgery" OR "urological surgery" OR "thoracic surgery" OR "vascular surgery" OR "breast surgery" OR "head and neck surgery" OR "otolaryngologic surgery" OR "otorhinolaryngologic surgery" OR "plastic surgery" OR "transplant surgery") AND TITLE-ABS-KEY("postoperative nausea and vomiting" OR "post-operative nausea and vomiting" OR "postoperative nausea or vomiting" OR "post-operative nausea or vomiting" OR PONV OR "postoperative nausea" OR "post-operative nausea" OR "postoperative vomiting" OR "post-operative vomiting" OR "postoperative emesis" OR "post-operative emesis" OR "postanesthesia nausea" OR "postanaesthesia nausea" OR "post-anesthesia nausea" OR "post-anaesthesia nausea" OR "postanesthetic nausea" OR "postanaesthetic nausea" OR "postanesthesia vomiting" OR "postanaesthesia vomiting" OR "post-anesthesia vomiting" OR "post-anaesthesia vomiting" OR "postanesthetic vomiting" OR "postanaesthetic vomiting" OR ((postoperativ* OR post-operativ* OR postanesthe* OR post-anesthe* OR postanaesthe* OR post-anaesthe*) W/3 (nausea OR vomit* OR emesis OR retching)) OR hypotens* OR "intraoperative hypotension" OR "intra-operative hypotension" OR "perioperative hypotension" OR "peri-operative hypotension" OR "postoperative hypotension" OR "post-operative hypotension" OR "postanesthesia care unit hypotension" OR "postanaesthesia care unit hypotension" OR "post-anesthesia care unit hypotension" OR "post-anaesthesia care unit hypotension" OR "PACU hypotension" OR "postinduction hypotension" OR "post-induction hypotension" OR "post induction hypotension" OR "hypotension after induction" OR "hypotension following induction" OR "anesthesia-induced hypotension" OR "anaesthesia-induced hypotension" OR "anesthetic-induced hypotension" OR "anaesthetic-induced hypotension" OR "anesthesia-related hypotension" OR "anaesthesia-related hypotension" OR "arterial hypotension" OR "low blood pressure" OR "low arterial pressure" OR "low mean arterial pressure" OR "decreased mean arterial pressure" OR "mean arterial pressure decrease" OR "hemodynamic instability" OR "haemodynamic instability" OR "circulatory instability" OR "cardiovascular instability" OR "blood pressure instability" OR "hypotension prediction index" OR HPI OR vasopleg* OR ((blood OR arterial OR hemodynamic OR haemodynamic) W/3 (pressure OR instability OR hypotens*)) OR (((ICU OR ICUs OR "intensive care" OR "intensive care unit*" OR "critical care" OR "critical care unit*") W/5 ("length of stay" OR stay OR stays OR prolonged OR prolong* OR extended OR extend* OR longer OR long OR duration OR admission OR admissions OR transfer OR transfers OR requirement OR requirements OR need OR needs OR utilization OR utilisation OR occupancy)) OR "prolonged ICU stay" OR "prolonged intensive care stay" OR "extended ICU stay" OR "extended intensive care stay" OR "long ICU stay" OR "ICU length of stay" OR "intensive care length of stay" OR "critical care length of stay" OR "ICU admission" OR "intensive care admission" OR "critical care admission" OR "ICU requirement" OR "intensive care requirement" OR "need for intensive care" OR "requirement for intensive care" OR "unplanned ICU admission" OR "unexpected ICU admission" OR "unplanned intensive care admission" OR "unexpected intensive care admission" OR "postoperative ICU admission" OR "post-operative ICU admission" OR "postoperative intensive care admission" OR "post-operative intensive care admission") OR mortality OR death OR deaths OR fatal* OR "in-hospital mortality" OR "in hospital mortality" OR "hospital mortality" OR "inpatient mortality" OR "in-patient mortality" OR "perioperative mortality" OR "peri-operative mortality" OR "postoperative mortality" OR "post-operative mortality" OR "operative mortality" OR "surgical mortality" OR "in-hospital death" OR "in hospital death" OR "hospital death" OR "inpatient death" OR "postoperative death" OR "post-operative death" OR "perioperative death" OR "peri-operative death" OR "operative death" OR "surgical death" OR "death after surgery" OR "death following surgery" OR "30-day mortality" OR "30 day mortality" OR "thirty-day mortality" OR "short-term mortality" OR "short term mortality" OR "all-cause mortality" OR "all cause mortality" OR ((hospital OR inpatient OR perioperativ* OR postoperativ* OR operativ* OR surgical) W/3 (mortality OR death OR deaths OR fatal*)) OR "neurological complication*" OR "neurologic complication*" OR "neurological adverse event*" OR "neurologic adverse event*" OR "neurological outcome*" OR "neurologic outcome*" OR "neurological morbidity" OR "neurologic morbidity" OR "postoperative neurological complication*" OR "postoperative neurologic complication*" OR "post-operative neurological complication*" OR "post-operative neurologic complication*" OR "perioperative neurological complication*" OR "perioperative neurologic complication*" OR "peri-operative neurological complication*" OR "peri-operative neurologic complication*" OR "central nervous system complication*" OR "CNS complication*" OR "perioperative neurocognitive disorder*" OR "peri-operative neurocognitive disorder*" OR "postoperative neurocognitive disorder*" OR "post-operative neurocognitive disorder*" OR "postoperative cognitive dysfunction" OR "post-operative cognitive dysfunction" OR POCD OR "postoperative cognitive decline" OR "post-operative cognitive decline" OR "cognitive decline" OR "cognitive deterioration" OR "cognitive impairment" OR "postoperative cognitive impairment" OR "post-operative cognitive impairment" OR "postoperative delirium" OR "post-operative delirium" OR "emergence delirium" OR "emergence agitation" OR "perioperative delirium" OR "peri-operative delirium" OR "postoperative stroke" OR "post-operative stroke" OR "perioperative stroke" OR "peri-operative stroke" OR "cerebrovascular accident*" OR "cerebrovascular complication*" OR "cerebral ischemia" OR "cerebral ischaemia" OR "brain ischemia" OR "brain ischaemia" OR "cerebral infarction" OR "brain infarction" OR "cerebral hypoperfusion" OR "brain hypoperfusion" OR "brain injury" OR "cerebral injury" OR encephalopath* OR seizure* OR convulsion* OR coma OR "altered consciousness" OR "loss of consciousness" OR "neurological deficit*" OR "neurologic deficit*" OR "neurological dysfunction" OR "neurologic dysfunction" OR ((postoperativ* OR perioperativ* OR surgical OR surgery) W/5 (neurolog* OR neurocognit* OR delirium OR stroke OR encephalopath* OR seizure* OR coma OR "cognitive dysfunction" OR "cognitive impairment"))) AND TITLE-ABS-KEY(predict* OR prognos* OR forecast* OR estimat* OR "risk prediction" OR "risk-prediction" OR "risk estimation" OR "risk-estimation" OR "risk model*" OR "prediction model*" OR "predictive model*" OR "prognostic model*" OR "prognosis model*" OR "risk score*" OR "prediction score*" OR "predictive score*" OR "prognostic score*" OR "risk stratification" OR "risk-stratification" OR "risk assessment" OR "individual risk" OR "individualized risk" OR "individualised risk" OR "personalized risk" OR "personalised risk" OR "clinical prediction" OR "clinical predictive model*" OR "prediction tool*" OR "predictive tool*" OR "prognostic tool*" OR "prediction rule*" OR "clinical prediction rule*" OR "clinical decision rule*" OR "risk calculator*" OR "prediction calculator*" OR classifier* OR classification OR discriminat* OR calibrat* OR validat* OR "internal validation" OR "external validation" OR "temporal validation" OR "geographical validation" OR "geographic validation" OR "prospective validation" OR "retrospective validation" OR "cross-validation" OR "cross validation" OR "k-fold cross-validation" OR "k fold cross validation" OR bootstrap* OR "model development" OR "model derivation" OR "model construction" OR "model building" OR "model training" OR "model testing" OR "model evaluation" OR "model assessment" OR "model comparison" OR "model performance" OR "predictive performance" OR "prediction performance" OR "prognostic performance" OR "classification performance" OR "diagnostic performance" OR accuracy OR sensitivity OR specificity OR discrimination OR calibration OR AUROC OR AUC OR "AUC-ROC" OR "area under the curve" OR "area under receiver operating characteristic curve" OR "area under the receiver operating characteristic curve" OR "receiver operating characteristic" OR "receiver-operating characteristic" OR "ROC curve" OR "ROC analysis" OR "c-statistic" OR "c statistic" OR "concordance statistic" OR "concordance index" OR "c-index" OR "Brier score" OR "F1 score" OR "F-score" OR precision OR recall OR "positive predictive value" OR "negative predictive value" OR PPV OR NPV OR "likelihood ratio" OR "decision curve" OR "decision-curve analysis" OR "decision curve analysis" OR "net benefit" OR "clinical utility" OR "early warning" OR "early-warning system*" OR "warning system*" OR "probability estimation" OR "probability prediction" OR "outcome prediction" OR "event prediction" OR "complication prediction")
Web of Science	TS=("artificial intelligence" OR "artificial-intelligence" OR AI OR "machine intelligence" OR "computational intelligence" OR "intelligent computing" OR "machine learning" OR "machine-learning" OR ML OR "deep learning" OR "deep-learning" OR "representation learning" OR "supervised learning" OR "unsupervised learning" OR "semi-supervised learning" OR "semisupervised learning" OR "reinforcement learning" OR "transfer learning" OR "federated learning" OR "statistical learning" OR "automated learning" OR "automated machine learning" OR AutoML OR "predictive analytics" OR "data mining" OR "pattern recognition" OR "pattern analysis" OR "machine pattern recognition" OR "expert system*" OR "knowledge-based system*" OR "knowledge based system*" OR "intelligent system*" OR "clinical decision support system*" OR "computer-aided decision*" OR "computer aided decision*" OR "computer-assisted decision*" OR "computer assisted decision*" OR "artificial neural network*" OR "neural network*" OR ANN OR DNN OR CNN OR RNN OR LSTM OR GRU OR "deep neural network*" OR "convolutional neural network*" OR "recurrent neural network*" OR "recursive neural network*" OR "long short-term memory" OR "long short term memory" OR "gated recurrent unit*" OR "multilayer perceptron*" OR "multi-layer perceptron*" OR MLP OR "feedforward neural network*" OR "feed-forward neural network*" OR "backpropagation neural network*" OR "back-propagation neural network*" OR "probabilistic neural network*" OR "radial basis function network*" OR "self-organizing map*" OR "self organising map*" OR "transformer model*" OR "transformer network*" OR "attention-based model*" OR "attention based model*" OR "decision tree*" OR "classification tree*" OR "regression tree*" OR CART OR "C4.5" OR "C5.0" OR "random forest*" OR "balanced random forest*" OR "conditional random forest*" OR "conditional inference forest*" OR cforest OR "extremely randomized tree*" OR "extremely randomised tree*" OR "extra tree*" OR "extra-tree*" OR "gradient boosting" OR "gradient-boosting" OR "gradient boosting machine*" OR GBM OR GBDT OR "gradient boosting decision tree*" OR "extreme gradient boosting" OR XGBoost OR LightGBM OR "light gradient boosting machine*" OR CatBoost OR AdaBoost OR "adaptive boosting" OR "boosted tree*" OR "boosting algorithm*" OR bagging OR "bootstrap aggregating" OR stacking OR "stacked generalization" OR "stacked generalisation" OR "ensemble learning" OR "ensemble model*" OR "ensemble method*" OR "ensemble classifier*" OR "ensemble algorithm*" OR "voting classifier*" OR "support vector machine*" OR "support-vector machine*" OR "support vector classifier*" OR "support-vector classifier*" OR SVM OR SVC OR "kernel machine*" OR "kernel method*" OR "k-nearest neighbor*" OR "k nearest neighbor*" OR "k-nearest neighbour*" OR "k nearest neighbour*" OR KNN OR "nearest neighbor algorithm*" OR "nearest neighbour algorithm*" OR "nearest-neighbor classifier*" OR "nearest-neighbour classifier*" OR "naive Bayes" OR "naïve Bayes" OR "naive Bayesian" OR "naïve Bayesian" OR "Bayesian classifier*" OR "Bayes classifier*" OR "Bayesian network*" OR "Bayesian machine learning" OR "Bayesian additive regression tree*" OR BART OR "Gaussian naive Bayes" OR "Gaussian naïve Bayes" OR GNB OR "Gaussian process*" OR "linear discriminant analysis" OR LDA OR "quadratic discriminant analysis" OR QDA OR "discriminant analysis classifier*" OR "logistic model tree*" OR "fuzzy logic" OR "fuzzy system*" OR "fuzzy inference" OR "neuro-fuzzy" OR "neuro fuzzy" OR "genetic algorithm*" OR "evolutionary algorithm*" OR "evolutionary computation" OR "genetic programming" OR "particle swarm optimization" OR "particle swarm optimisation" OR "ant colony optimization" OR "ant colony optimisation" OR "synthetic minority oversampling technique" OR "synthetic minority over-sampling technique" OR SMOTE OR BSMOTE OR "borderline SMOTE" OR "borderline synthetic minority oversampling" OR "minority oversampling" OR "minority over-sampling" OR "random oversampling" OR "random over-sampling" OR "class imbalance" OR "class-imbalance" OR "imbalanced data" OR "imbalanced dataset*" OR "imbalanced learning" OR "cost-sensitive learning" OR "cost sensitive learning" OR "recursive feature elimination" OR RFE OR "feature selection algorithm*" OR "learning algorithm*" OR "classification algorithm*" OR "predictive algorithm*" OR "prediction algorithm*" OR "explainable artificial intelligence" OR "explainable AI" OR XAI OR SHAP OR "Shapley additive explanation*" OR LIME) AND TS=("general anesthesia" OR "general anaesthesia" OR "general anesthetic*" OR "general anaesthetic*" OR (general NEAR/2 (anesthe* OR anaesthe*)) OR anesthesiolog* OR anaesthesiolog* OR anesthesia OR anaesthesia OR anesthetic* OR anaesthetic* OR perioperativ* OR peri-operativ* OR perianesthe* OR peri-anesthe* OR perianaesthe* OR peri-anaesthe* OR preoperativ* OR pre-operativ* OR intraoperativ* OR intra-operativ* OR postoperativ* OR post-operativ* OR postanesthe* OR post-anesthe* OR postanaesthe* OR post-anaesthe* OR "post anesthesia care unit" OR "post anaesthesia care unit" OR "post-anesthesia care unit" OR "post-anaesthesia care unit" OR "postanesthesia care unit" OR "postanaesthesia care unit" OR PACU OR "anesthesia recovery" OR "anaesthesia recovery" OR "recovery from anesthesia" OR "recovery from anaesthesia" OR "anesthetic recovery" OR "anaesthetic recovery" OR "recovery room*" OR "operating room*" OR "operating theatre*" OR "operating theater*" OR "surgical theatre*" OR "surgical theater*" OR surger* OR surgical OR operativ* OR "surgical patient*" OR "operative patient*" OR "surgical population*" OR "surgical procedure*" OR "operative procedure*" OR "surgical operation*" OR "ambulatory surgery" OR "ambulatory surgical procedure*" OR "outpatient surgery" OR "outpatient surgical procedure*" OR "day surgery" OR "day-case surgery" OR "day case surgery" OR "day-care surgery" OR "day care surgery" OR "inpatient surgery" OR "in-patient surgery" OR "major surgery" OR "major surgical procedure*" OR "elective surgery" OR "elective surgical procedure*" OR "emergency surgery" OR "emergency surgical procedure*" OR "urgent surgery" OR "noncardiac surgery" OR "non-cardiac surgery" OR "cardiac surgery" OR "cardiovascular surgery" OR "general surgery" OR "abdominal surgery" OR "gastrointestinal surgery" OR "laparoscopic surgery" OR "laparoscopic procedure*" OR "orthopedic surgery" OR "orthopaedic surgery" OR neurosurgery OR "neurological surgery" OR "gynecologic surgery" OR "gynaecologic surgery" OR "urologic surgery" OR "urological surgery" OR "thoracic surgery" OR "vascular surgery" OR "breast surgery" OR "head and neck surgery" OR "otolaryngologic surgery" OR "otorhinolaryngologic surgery" OR "plastic surgery" OR "transplant surgery") AND TS=("postoperative nausea and vomiting" OR "post-operative nausea and vomiting" OR "postoperative nausea or vomiting" OR "post-operative nausea or vomiting" OR PONV OR "postoperative nausea" OR "post-operative nausea" OR "postoperative vomiting" OR "post-operative vomiting" OR "postoperative emesis" OR "post-operative emesis" OR "postanesthesia nausea" OR "postanaesthesia nausea" OR "post-anesthesia nausea" OR "post-anaesthesia nausea" OR "postanesthetic nausea" OR "postanaesthetic nausea" OR "postanesthesia vomiting" OR "postanaesthesia vomiting" OR "post-anesthesia vomiting" OR "post-anaesthesia vomiting" OR "postanesthetic vomiting" OR "postanaesthetic vomiting" OR ((postoperativ* OR post-operativ* OR postanesthe* OR post-anesthe* OR postanaesthe* OR post-anaesthe*) NEAR/3 (nausea OR vomit* OR emesis OR retching)) OR hypotens* OR "intraoperative hypotension" OR "intra-operative hypotension" OR "perioperative hypotension" OR "peri-operative hypotension" OR "postoperative hypotension" OR "post-operative hypotension" OR "postanesthesia care unit hypotension" OR "postanaesthesia care unit hypotension" OR "post-anesthesia care unit hypotension" OR "post-anaesthesia care unit hypotension" OR "PACU hypotension" OR "postinduction hypotension" OR "post-induction hypotension" OR "post induction hypotension" OR "hypotension after induction" OR "hypotension following induction" OR "anesthesia-induced hypotension" OR "anaesthesia-induced hypotension" OR "anesthetic-induced hypotension" OR "anaesthetic-induced hypotension" OR "anesthesia-related hypotension" OR "anaesthesia-related hypotension" OR "arterial hypotension" OR "low blood pressure" OR "low arterial pressure" OR "low mean arterial pressure" OR "decreased mean arterial pressure" OR "mean arterial pressure decrease" OR "hemodynamic instability" OR "haemodynamic instability" OR "circulatory instability" OR "cardiovascular instability" OR "blood pressure instability" OR "hypotension prediction index" OR HPI OR vasopleg* OR ((blood OR arterial OR hemodynamic OR haemodynamic) NEAR/3 (pressure OR instability OR hypotens*)) OR ((ICU OR ICUs OR "intensive care" OR "intensive care unit*" OR "critical care" OR "critical care unit*") NEAR/5 ("length of stay" OR stay OR stays OR prolonged OR prolong* OR extended OR extend* OR longer OR long OR duration OR admission OR admissions OR transfer OR transfers OR requirement OR requirements OR need OR needs OR utilization OR utilisation OR occupancy)) OR "prolonged ICU stay" OR "prolonged intensive care stay" OR "extended ICU stay" OR "extended intensive care stay" OR "long ICU stay" OR "ICU length of stay" OR "intensive care length of stay" OR "critical care length of stay" OR "ICU admission" OR "intensive care admission" OR "critical care admission" OR "ICU requirement" OR "intensive care requirement" OR "need for intensive care" OR "requirement for intensive care" OR "unplanned ICU admission" OR "unexpected ICU admission" OR "unplanned intensive care admission" OR "unexpected intensive care admission" OR "postoperative ICU admission" OR "post-operative ICU admission" OR "postoperative intensive care admission" OR "post-operative intensive care admission" OR mortality OR death OR deaths OR fatal* OR "in-hospital mortality" OR "in hospital mortality" OR "hospital mortality" OR "inpatient mortality" OR "in-patient mortality" OR "perioperative mortality" OR "peri-operative mortality" OR "postoperative mortality" OR "post-operative mortality" OR "operative mortality" OR "surgical mortality" OR "in-hospital death" OR "in hospital death" OR "hospital death" OR "inpatient death" OR "postoperative death" OR "post-operative death" OR "perioperative death" OR "peri-operative death" OR "operative death" OR "surgical death" OR "death after surgery" OR "death following surgery" OR "30-day mortality" OR "30 day mortality" OR "thirty-day mortality" OR "short-term mortality" OR "short term mortality" OR "all-cause mortality" OR "all cause mortality" OR ((hospital OR inpatient OR perioperativ* OR postoperativ* OR operativ* OR surgical) NEAR/3 (mortality OR death OR deaths OR fatal*)) OR "neurological complication*" OR "neurologic complication*" OR "neurological adverse event*" OR "neurologic adverse event*" OR "neurological outcome*" OR "neurologic outcome*" OR "neurological morbidity" OR "neurologic morbidity" OR "postoperative neurological complication*" OR "postoperative neurologic complication*" OR "post-operative neurological complication*" OR "post-operative neurologic complication*" OR "perioperative neurological complication*" OR "perioperative neurologic complication*" OR "peri-operative neurological complication*" OR "peri-operative neurologic complication*" OR "central nervous system complication*" OR "CNS complication*" OR "perioperative neurocognitive disorder*" OR "peri-operative neurocognitive disorder*" OR "postoperative neurocognitive disorder*" OR "post-operative neurocognitive disorder*" OR "postoperative cognitive dysfunction" OR "post-operative cognitive dysfunction" OR POCD OR "postoperative cognitive decline" OR "post-operative cognitive decline" OR "cognitive decline" OR "cognitive deterioration" OR "cognitive impairment" OR "postoperative cognitive impairment" OR "post-operative cognitive impairment" OR "postoperative delirium" OR "post-operative delirium" OR "emergence delirium" OR "emergence agitation" OR "perioperative delirium" OR "peri-operative delirium" OR "postoperative stroke" OR "post-operative stroke" OR "perioperative stroke" OR "peri-operative stroke" OR "cerebrovascular accident*" OR "cerebrovascular complication*" OR "cerebral ischemia" OR "cerebral ischaemia" OR "brain ischemia" OR "brain ischaemia" OR "cerebral infarction" OR "brain infarction" OR "cerebral hypoperfusion" OR "brain hypoperfusion" OR "brain injury" OR "cerebral injury" OR encephalopath* OR seizure* OR convulsion* OR coma OR "altered consciousness" OR "loss of consciousness" OR "neurological deficit*" OR "neurologic deficit*" OR "neurological dysfunction" OR "neurologic dysfunction" OR ((postoperativ* OR perioperativ* OR surgical OR surgery) NEAR/5 (neurolog* OR neurocognit* OR delirium OR stroke OR encephalopath* OR seizure* OR coma OR "cognitive dysfunction" OR "cognitive impairment"))) AND TS=(predict* OR prognos* OR forecast* OR estimat* OR "risk prediction" OR "risk-prediction" OR "risk estimation" OR "risk-estimation" OR "risk model*" OR "prediction model*" OR "predictive model*" OR "prognostic model*" OR "prognosis model*" OR "risk score*" OR "prediction score*" OR "predictive score*" OR "prognostic score*" OR "risk stratification" OR "risk-stratification" OR "risk assessment" OR "individual risk" OR "individualized risk" OR "individualised risk" OR "personalized risk" OR "personalised risk" OR "clinical prediction" OR "clinical predictive model*" OR "prediction tool*" OR "predictive tool*" OR "prognostic tool*" OR "prediction rule*" OR "clinical prediction rule*" OR "clinical decision rule*" OR "risk calculator*" OR "prediction calculator*" OR classifier* OR classification OR discriminat* OR calibrat* OR validat* OR "internal validation" OR "external validation" OR "temporal validation" OR "geographical validation" OR "geographic validation" OR "prospective validation" OR "retrospective validation" OR "cross-validation" OR "cross validation" OR "k-fold cross-validation" OR "k fold cross validation" OR bootstrap* OR "model development" OR "model derivation" OR "model construction" OR "model building" OR "model training" OR "model testing" OR "model evaluation" OR "model assessment" OR "model comparison" OR "model performance" OR "predictive performance" OR "prediction performance" OR "prognostic performance" OR "classification performance" OR "diagnostic performance" OR accuracy OR sensitivity OR specificity OR discrimination OR calibration OR AUROC OR AUC OR "AUC-ROC" OR "area under the curve" OR "area under receiver operating characteristic curve" OR "area under the receiver operating characteristic curve" OR "receiver operating characteristic" OR "receiver-operating characteristic" OR "ROC curve" OR "ROC analysis" OR "c-statistic" OR "c statistic" OR "concordance statistic" OR "concordance index" OR "c-index" OR "Brier score" OR "F1 score" OR "F-score" OR precision OR recall OR "positive predictive value" OR "negative predictive value" OR PPV OR NPV OR "likelihood ratio" OR "decision curve" OR "decision-curve analysis" OR "decision curve analysis" OR "net benefit" OR "clinical utility" OR "early warning" OR "early-warning system*" OR "warning system*" OR "probability estimation" OR "probability prediction" OR "outcome prediction" OR "event prediction" OR "complication prediction")
Cochrane library	([mh "Artificial Intelligence"] OR [mh "Machine Learning"] OR [mh "Deep Learning"] OR [mh "Neural Networks, Computer"] OR [mh "Data Mining"] OR [mh "Decision Trees"] OR [mh "Algorithms"] OR [mh "Pattern Recognition, Automated"] OR [mh "Expert Systems"] OR [mh "Fuzzy Logic"] OR [mh "Genetic Algorithms"] OR [mh "Prediction Methods, Machine"] OR [mh "Predictive Learning Models"] OR ("artificial intelligence" OR artificial-intelligence OR AI OR "machine intelligence" OR "computational intelligence" OR "intelligent computing" OR "machine learning" OR machine-learning OR ML OR "deep learning" OR deep-learning OR "representation learning" OR "supervised learning" OR "unsupervised learning" OR "semi-supervised learning" OR "semisupervised learning" OR "self-supervised learning" OR "reinforcement learning" OR "transfer learning" OR "federated learning" OR "statistical learning" OR "automated learning" OR "automated machine learning" OR AutoML OR "predictive analytics" OR "data mining" OR "pattern recognition" OR "pattern analysis" OR "machine pattern recognition" OR (expert NEXT system*) OR (knowledge-based NEXT system*) OR (knowledge NEXT based NEXT system*) OR (intelligent NEXT system*) OR (clinical NEXT decision NEXT support NEXT system*) OR (computer-aided NEXT decision*) OR (computer NEXT aided NEXT decision*) OR (computer-assisted NEXT decision*) OR (computer NEXT assisted NEXT decision*) OR (artificial NEXT neural NEXT network*) OR (neural NEXT network*) OR ANN OR DNN OR CNN OR RNN OR LSTM OR GRU OR CNNRNN OR (deep NEXT neural NEXT network*) OR (convolutional NEXT neural NEXT network*) OR (recurrent NEXT neural NEXT network*) OR (recursive NEXT neural NEXT network*) OR "long short-term memory" OR "long short term memory" OR (gated NEXT recurrent NEXT unit*) OR (multilayer NEXT perceptron*) OR (multi-layer NEXT perceptron*) OR MLP OR (feedforward NEXT neural NEXT network*) OR (feed-forward NEXT neural NEXT network*) OR (backpropagation NEXT neural NEXT network*) OR (back-propagation NEXT neural NEXT network*) OR (probabilistic NEXT neural NEXT network*) OR (radial NEXT basis NEXT function NEXT network*) OR (self-organizing NEXT map*) OR (self NEXT organizing NEXT map*) OR (self-organising NEXT map*) OR (self NEXT organising NEXT map*) OR (deep NEXT belief NEXT network*) OR (restricted NEXT Boltzmann NEXT machine*) OR (extreme NEXT learning NEXT machine*) OR ELM OR (graph NEXT neural NEXT network*) OR GNN OR (transformer NEXT model*) OR (transformer NEXT network*) OR (attention-based NEXT model*) OR (attention NEXT based NEXT model*) OR autoencoder* OR (deep NEXT autoencoder*) OR (variational NEXT autoencoder*) OR (decision NEXT tree*) OR (classification NEXT tree*) OR (regression NEXT tree*) OR CART OR "C4.5" OR "C5.0" OR (random NEXT forest*) OR (balanced NEXT random NEXT forest*) OR (conditional NEXT random NEXT forest*) OR (conditional NEXT inference NEXT forest*) OR cforest OR (extremely NEXT randomized NEXT tree*) OR (extremely NEXT randomised NEXT tree*) OR (extra NEXT tree*) OR (extra-tree*) OR "gradient boosting" OR gradient-boosting OR (gradient NEXT boosting NEXT machine*) OR GBM OR GBDT OR (gradient NEXT boosting NEXT decision NEXT tree*) OR (gradient NEXT boosted NEXT tree*) OR (extreme NEXT gradient NEXT boosting) OR XGBoost OR LightGBM OR (light NEXT gradient NEXT boosting NEXT machine*) OR CatBoost OR AdaBoost OR (adaptive NEXT boosting) OR (boosted NEXT tree*) OR (boosting NEXT algorithm*) OR bagging OR "bootstrap aggregating" OR stacking OR "stacked generalization" OR "stacked generalisation" OR "ensemble learning" OR (ensemble NEXT model*) OR (ensemble NEXT method*) OR (ensemble NEXT classifier*) OR (ensemble NEXT algorithm*) OR (voting NEXT classifier*) OR (blending NEXT model*) OR (support NEXT vector NEXT machine*) OR (support-vector NEXT machine*) OR (support NEXT vector NEXT classifier*) OR (support-vector NEXT classifier*) OR SVM OR SVC OR (kernel NEXT machine*) OR (kernel NEXT method*) OR (k-nearest NEXT neighbor*) OR (k NEXT nearest NEXT neighbor*) OR (k-nearest NEXT neighbour*) OR (k NEXT nearest NEXT neighbour*) OR KNN OR (nearest NEXT neighbor NEXT algorithm*) OR (nearest NEXT neighbour NEXT algorithm*) OR (nearest-neighbor NEXT classifier*) OR (nearest-neighbour NEXT classifier*) OR "naive Bayes" OR "naïve Bayes" OR "naive Bayesian" OR "naïve Bayesian" OR (Bayesian NEXT classifier*) OR (Bayes NEXT classifier*) OR (Bayesian NEXT network*) OR "Bayesian machine learning" OR (Bayesian NEXT additive NEXT regression NEXT tree*) OR BART OR "Gaussian naive Bayes" OR "Gaussian naïve Bayes" OR GNB OR (Gaussian NEXT process*) OR "linear discriminant analysis" OR LDA OR "quadratic discriminant analysis" OR QDA OR (discriminant NEXT analysis NEXT classifier*) OR (logistic NEXT model NEXT tree*) OR "fuzzy logic" OR (fuzzy NEXT system*) OR (fuzzy NEXT inference) OR neuro-fuzzy OR (neuro NEXT fuzzy) OR (genetic NEXT algorithm*) OR (evolutionary NEXT algorithm*) OR "evolutionary computation" OR "genetic programming" OR "particle swarm optimization" OR "particle swarm optimisation" OR "ant colony optimization" OR "ant colony optimisation" OR "synthetic minority oversampling technique" OR "synthetic minority over-sampling technique" OR SMOTE OR BSMOTE OR Borderline-SMOTE OR "borderline SMOTE" OR "borderline synthetic minority oversampling" OR "minority oversampling" OR "minority over-sampling" OR "random oversampling" OR "random over-sampling" OR "adaptive synthetic sampling" OR ADASYN OR "class imbalance" OR class-imbalance OR "imbalanced data" OR (imbalanced NEXT dataset*) OR "imbalanced learning" OR "cost-sensitive learning" OR "cost sensitive learning" OR "recursive feature elimination" OR RFE OR (feature NEXT selection NEXT algorithm*) OR (learning NEXT algorithm*) OR (classification NEXT algorithm*) OR (predictive NEXT algorithm*) OR (prediction NEXT algorithm*) OR (machine-learned NEXT model*) OR (machine NEXT learned NEXT model*) OR "explainable artificial intelligence" OR "explainable AI" OR XAI OR SHAP OR "Shapley additive explanations" OR "Shapley additive explanation" OR LIME):ti,ab,kw) AND ([mh "Anesthesia, General"] OR [mh "Anesthesiology"] OR [mh "Perioperative Care"] OR [mh "Perioperative Period"] OR [mh "Preoperative Care"] OR [mh "Intraoperative Care"] OR [mh "Postoperative Care"] OR [mh "Postoperative Period"] OR [mh "Anesthesia Recovery Period"] OR [mh "Surgical Procedures, Operative"] OR [mh "Elective Surgical Procedures"] OR [mh "Ambulatory Surgical Procedures"] OR [mh "Operating Rooms"] OR ("general anesthesia" OR "general anaesthesia" OR (general NEXT anesthe*) OR (general NEXT anaesthe*) OR (general NEXT anesthetic*) OR (general NEXT anaesthetic*) OR anesthesiolog* OR anaesthesiolog* OR anesthesia OR anaesthesia OR anesthetic* OR anaesthetic* OR perioperativ* OR peri-operativ* OR perianesthe* OR peri-anesthe* OR perianaesthe* OR peri-anaesthe* OR preoperativ* OR pre-operativ* OR intraoperativ* OR intra-operativ* OR postoperativ* OR post-operativ* OR postanesthe* OR post-anesthe* OR postanaesthe* OR post-anaesthe* OR "post anesthesia care unit" OR "post anaesthesia care unit" OR "post-anesthesia care unit" OR "post-anaesthesia care unit" OR "postanesthesia care unit" OR "postanaesthesia care unit" OR PACU OR "anesthesia recovery" OR "anaesthesia recovery" OR "recovery from anesthesia" OR "recovery from anaesthesia" OR "anesthetic recovery" OR "anaesthetic recovery" OR (recovery NEXT room*) OR (operating NEXT room*) OR (operating NEXT theatre*) OR (operating NEXT theater*) OR (surgical NEXT theatre*) OR (surgical NEXT theater*) OR surger* OR surgical OR operativ* OR (surgical NEXT patient*) OR (operative NEXT patient*) OR (surgical NEXT population*) OR (surgical NEXT procedure*) OR (operative NEXT procedure*) OR (surgical NEXT operation*) OR "ambulatory surgery" OR (ambulatory NEXT surgical NEXT procedure*) OR "outpatient surgery" OR (outpatient NEXT surgical NEXT procedure*) OR "day surgery" OR "day-case surgery" OR "day case surgery" OR "day-care surgery" OR "day care surgery" OR "inpatient surgery" OR "in-patient surgery" OR "major surgery" OR (major NEXT surgical NEXT procedure*) OR "elective surgery" OR (elective NEXT surgical NEXT procedure*) OR "emergency surgery" OR (emergency NEXT surgical NEXT procedure*) OR "urgent surgery" OR "noncardiac surgery" OR "non-cardiac surgery" OR "cardiac surgery" OR "cardiovascular surgery" OR "general surgery" OR "abdominal surgery" OR "gastrointestinal surgery" OR "digestive surgery" OR "laparoscopic surgery" OR (laparoscopic NEXT procedure*) OR "orthopedic surgery" OR "orthopaedic surgery" OR neurosurgery OR "neurological surgery" OR "gynecologic surgery" OR "gynaecologic surgery" OR "obstetric surgery" OR "urologic surgery" OR "urological surgery" OR "thoracic surgery" OR "vascular surgery" OR "breast surgery" OR "head and neck surgery" OR "otolaryngologic surgery" OR "otorhinolaryngologic surgery" OR "plastic surgery" OR "transplant surgery" OR "oral surgery" OR "maxillofacial surgery" OR "ophthalmic surgery" OR "spinal surgery" OR "spine surgery" OR "robotic surgery"):ti,ab,kw) AND (([mh "Postoperative Nausea and Vomiting"] OR ("postoperative nausea and vomiting" OR "post-operative nausea and vomiting" OR "postoperative nausea or vomiting" OR "post-operative nausea or vomiting" OR PONV OR "postoperative nausea" OR "post-operative nausea" OR "postoperative vomiting" OR "post-operative vomiting" OR "postoperative emesis" OR "post-operative emesis" OR "postanesthesia nausea" OR "postanaesthesia nausea" OR "post-anesthesia nausea" OR "post-anaesthesia nausea" OR "postanesthetic nausea" OR "postanaesthetic nausea" OR "postanesthesia vomiting" OR "postanaesthesia vomiting" OR "post-anesthesia vomiting" OR "post-anaesthesia vomiting" OR "postanesthetic vomiting" OR "postanaesthetic vomiting" OR ((postoperativ* OR post-operativ* OR postanesthe* OR post-anesthe* OR postanaesthe* OR post-anaesthe*) NEAR/3 (nausea OR vomit* OR emesis OR retching))):ti,ab,kw) OR ([mh "Hypotension"] OR (hypotens* OR "intraoperative hypotension" OR "intra-operative hypotension" OR "perioperative hypotension" OR "peri-operative hypotension" OR "postoperative hypotension" OR "post-operative hypotension" OR "postanesthesia care unit hypotension" OR "postanaesthesia care unit hypotension" OR "post-anesthesia care unit hypotension" OR "post-anaesthesia care unit hypotension" OR "PACU hypotension" OR "postinduction hypotension" OR "post-induction hypotension" OR "post induction hypotension" OR "hypotension after induction" OR "hypotension following induction" OR "anesthesia-induced hypotension" OR "anaesthesia-induced hypotension" OR "anesthetic-induced hypotension" OR "anaesthetic-induced hypotension" OR "anesthesia-related hypotension" OR "anaesthesia-related hypotension" OR "arterial hypotension" OR "low blood pressure" OR "low arterial pressure" OR "low mean arterial pressure" OR "decreased mean arterial pressure" OR "mean arterial pressure decrease" OR "hemodynamic instability" OR "haemodynamic instability" OR "circulatory instability" OR "cardiovascular instability" OR "blood pressure instability" OR "hypotension prediction index" OR HPI OR vasopleg* OR ((blood OR arterial OR hemodynamic OR haemodynamic) NEAR/3 (pressure OR instability OR hypotens*))):ti,ab,kw) OR (([mh "Intensive Care Units"] OR [mh "Critical Care"]) AND ([mh "Length of Stay"] OR ((ICU OR ICUs OR "intensive care" OR (intensive NEXT care NEXT unit*) OR "critical care" OR (critical NEXT care NEXT unit*)) NEAR/5 ("length of stay" OR stay OR stays OR prolonged OR prolong* OR extended OR extend* OR longer OR long OR duration OR admission OR admissions OR transfer OR transfers OR requirement OR requirements OR need OR needs OR utilization OR utilisation OR occupancy)) OR "prolonged ICU stay" OR "prolonged intensive care stay" OR "extended ICU stay" OR "extended intensive care stay" OR "long ICU stay" OR "ICU length of stay" OR "intensive care length of stay" OR "critical care length of stay" OR "ICU admission" OR "intensive care admission" OR "critical care admission" OR "ICU requirement" OR "intensive care requirement" OR "need for intensive care" OR "requirement for intensive care" OR "unplanned ICU admission" OR "unexpected ICU admission" OR "unplanned intensive care admission" OR "unexpected intensive care admission" OR "postoperative ICU admission" OR "post-operative ICU admission" OR "postoperative intensive care admission" OR "post-operative intensive care admission"):ti,ab,kw) OR ([mh "Mortality"] OR [mh "Hospital Mortality"] OR [mh "Fatal Outcome"] OR (mortality OR death OR deaths OR fatal* OR "in-hospital mortality" OR "in hospital mortality" OR "hospital mortality" OR "inpatient mortality" OR "in-patient mortality" OR "perioperative mortality" OR "peri-operative mortality" OR "postoperative mortality" OR "post-operative mortality" OR "operative mortality" OR "surgical mortality" OR "in-hospital death" OR "in hospital death" OR "hospital death" OR "inpatient death" OR "postoperative death" OR "post-operative death" OR "perioperative death" OR "peri-operative death" OR "operative death" OR "surgical death" OR "death after surgery" OR "death following surgery" OR "30-day mortality" OR "30 day mortality" OR "thirty-day mortality" OR "short-term mortality" OR "short term mortality" OR "all-cause mortality" OR "all cause mortality" OR ((hospital OR inpatient OR perioperativ* OR postoperativ* OR operativ* OR surgical) NEAR/3 (mortality OR death OR deaths OR fatal*))):ti,ab,kw) OR ([mh "Neurologic Manifestations"] OR [mh "Nervous System Diseases"] OR [mh "Postoperative Cognitive Complications"] OR [mh "Cognitive Dysfunction"] OR [mh "Delirium"] OR [mh "Emergence Delirium"] OR [mh "Stroke"] OR [mh "Cerebrovascular Disorders"] OR [mh "Brain Ischemia"] OR [mh "Cerebral Infarction"] OR [mh "Seizures"] OR [mh "Coma"] OR ("neurological complication" OR (neurological NEXT complication*) OR "neurologic complication" OR (neurologic NEXT complication*) OR (neurological NEXT adverse NEXT event*) OR (neurologic NEXT adverse NEXT event*) OR (neurological NEXT outcome*) OR (neurologic NEXT outcome*) OR "neurological morbidity" OR "neurologic morbidity" OR (postoperative NEXT neurological NEXT complication*) OR (postoperative NEXT neurologic NEXT complication*) OR (post-operative NEXT neurological NEXT complication*) OR (post-operative NEXT neurologic NEXT complication*) OR (perioperative NEXT neurological NEXT complication*) OR (perioperative NEXT neurologic NEXT complication*) OR (peri-operative NEXT neurological NEXT complication*) OR (peri-operative NEXT neurologic NEXT complication*) OR (central NEXT nervous NEXT system NEXT complication*) OR (CNS NEXT complication*) OR (perioperative NEXT neurocognitive NEXT disorder*) OR (peri-operative NEXT neurocognitive NEXT disorder*) OR (postoperative NEXT neurocognitive NEXT disorder*) OR (post-operative NEXT neurocognitive NEXT disorder*) OR "postoperative cognitive dysfunction" OR "post-operative cognitive dysfunction" OR POCD OR "postoperative cognitive decline" OR "post-operative cognitive decline" OR "cognitive decline" OR "cognitive deterioration" OR "cognitive impairment" OR "postoperative cognitive impairment" OR "post-operative cognitive impairment" OR "postoperative delirium" OR "post-operative delirium" OR "emergence delirium" OR "emergence agitation" OR "perioperative delirium" OR "peri-operative delirium" OR "postoperative stroke" OR "post-operative stroke" OR "perioperative stroke" OR "peri-operative stroke" OR (cerebrovascular NEXT accident*) OR (cerebrovascular NEXT complication*) OR "cerebral ischemia" OR "cerebral ischaemia" OR "brain ischemia" OR "brain ischaemia" OR "cerebral infarction" OR "brain infarction" OR "cerebral hypoperfusion" OR "brain hypoperfusion" OR "brain injury" OR "cerebral injury" OR encephalopath* OR seizure* OR convulsion* OR coma OR "altered consciousness" OR "loss of consciousness" OR (neurological NEXT deficit*) OR (neurologic NEXT deficit*) OR "neurological dysfunction" OR "neurologic dysfunction" OR ((postoperativ* OR perioperativ* OR surgical OR surgery) NEAR/5 (neurolog* OR neurocognit* OR delirium OR stroke OR encephalopath* OR seizure* OR coma OR "cognitive dysfunction" OR "cognitive impairment"))):ti,ab,kw) OR ([mh "Postoperative Complications"] OR ((postoperativ* OR post-operativ* OR perioperativ* OR peri-operativ* OR surgical OR surgery) NEAR/4 (complication* OR "adverse outcome" OR "adverse outcomes" OR morbidity OR deterioration OR "clinical outcome" OR "clinical outcomes")) OR "major postoperative complication" OR (major NEXT postoperative NEXT complication*) OR "major perioperative complication" OR (major NEXT perioperative NEXT complication*) OR "postoperative adverse outcome" OR (postoperative NEXT adverse NEXT outcome*) OR "perioperative adverse outcome" OR (perioperative NEXT adverse NEXT outcome*)):ti,ab,kw)) AND ([mh "Risk Assessment"] OR [mh "Prognosis"] OR [mh "Predictive Value of Tests"] OR [mh "Models, Statistical"] OR [mh "ROC Curve"] OR [mh "Sensitivity and Specificity"] OR [mh "Nomograms"] OR (predict* OR prognos* OR forecast* OR estimat* OR "risk prediction" OR risk-prediction OR "risk estimation" OR risk-estimation OR (risk NEXT model*) OR (prediction NEXT model*) OR (predictive NEXT model*) OR (prognostic NEXT model*) OR (prognosis NEXT model*) OR (risk NEXT score*) OR (prediction NEXT score*) OR (predictive NEXT score*) OR (prognostic NEXT score*) OR "risk stratification" OR risk-stratification OR "risk assessment" OR "individual risk" OR "individualized risk" OR "individualised risk" OR "personalized risk" OR "personalised risk" OR "clinical prediction" OR (clinical NEXT predictive NEXT model*) OR (prediction NEXT tool*) OR (predictive NEXT tool*) OR (prognostic NEXT tool*) OR (prediction NEXT rule*) OR (clinical NEXT prediction NEXT rule*) OR (clinical NEXT decision NEXT rule*) OR (risk NEXT calculator*) OR (prediction NEXT calculator*) OR classifier* OR classification OR discriminat* OR calibrat* OR validat* OR "internal validation" OR "external validation" OR "temporal validation" OR "geographical validation" OR "geographic validation" OR "prospective validation" OR "retrospective validation" OR cross-validation OR "cross validation" OR k-fold OR (k NEXT fold NEXT cross-validation) OR bootstrap* OR "model development" OR "model derivation" OR "model construction" OR "model building" OR "model training" OR "model testing" OR "model evaluation" OR "model assessment" OR "model comparison" OR "model performance" OR "predictive performance" OR "prediction performance" OR "prognostic performance" OR "classification performance" OR "diagnostic performance" OR accuracy OR sensitivity OR specificity OR discrimination OR calibration OR AUROC OR AUC OR AUC-ROC OR "area under the curve" OR "area under receiver operating characteristic curve" OR "area under the receiver operating characteristic curve" OR "receiver operating characteristic" OR receiver-operating-characteristic OR "ROC curve" OR "ROC analysis" OR c-statistic OR "c statistic" OR "concordance statistic" OR "concordance index" OR c-index OR "Brier score" OR "F1 score" OR F-score OR precision OR recall OR "positive predictive value" OR "negative predictive value" OR PPV OR NPV OR "likelihood ratio" OR "decision curve" OR "decision-curve analysis" OR "decision curve analysis" OR "net benefit" OR "clinical utility" OR "early warning" OR (early-warning NEXT system*) OR (warning NEXT system*) OR "probability estimation" OR "probability prediction" OR "outcome prediction" OR "event prediction" OR "complication prediction"):ti,ab,kw)

Study Selection

All retrieved records were imported into Rayyan (Rayyan Systems Inc., Cambridge, MA), and duplicate records were removed electronically and manually. The deduplicated records were transferred to the Rayyan screening platform for screening. Two reviewers independently screened titles and abstracts against the eligibility criteria. Full texts of potentially relevant reports were subsequently retrieved and independently assessed by the same reviewers. Disagreements at either stage were resolved through discussion; when consensus could not be reached, a third reviewer adjudicated. Reasons for excluding studies after full-text assessment were documented. The study-selection process was presented using the PRISMA 2020 flow diagram.

When multiple publications used the same or overlapping study population, they were examined to determine whether they represented separate models, external validations, model updates, or duplicate reporting. Complementary reports were linked and treated as one study where appropriate, while model-specific information was extracted from the report providing the most complete data.

Data Extraction

Standardized and pilot-tested extraction form was developed with reference to the CHecklist for critical Appraisal and data extraction for systematic Reviews of prediction Modelling Studies (CHARMS) [16].

Two reviewers independently extracted the data, or one reviewer extracted the data and a second reviewer verified all entries against the original reports. Disagreements were resolved through consensus or consultation with a third reviewer. Data were extracted at both the study level and the individual-model level.

At the study level, the extracted information included the first author and publication year, country and number of participating centers, study design and data source, prospective or retrospective nature of data collection, participant eligibility criteria, number of participants and number experiencing the predicted outcome, age and other reported demographic characteristics, surgical specialty or type of surgery, inpatient, ambulatory, operating-room, or post-anesthesia care setting, type of anesthesia, predicted outcome, outcome definition and ascertainment method, prediction horizon, and duration of follow-up where applicable.

At the model level, information was extracted on the intended clinical purpose and point of prediction; whether the study involved model development, internal validation, external validation, model updating, or a combination of these; the model or algorithm name; candidate and final predictors; timing of predictor measurement; predictor domains, including demographic, clinical, laboratory, surgical, anesthetic, medication, physiological, and postoperative variables; methods used for feature selection or dimensionality reduction; methods used to manage class imbalance, including oversampling or synthetic minority oversampling techniques; handling of missing data; training, validation, and test-set sample sizes; data-splitting strategy; resampling methods, including cross-validation or bootstrapping; hyperparameter selection and model tuning where reported; type of internal, temporal, geographical, or external validation; comparator algorithms or conventional clinical risk scores; and availability of model equations, code, or implementation details.

Each distinct algorithm was treated as a separate model. Models evaluated at different prediction times were also extracted separately. Thus, early and delayed PONV models, hypotension predictions at 5, 10, and 15 minutes, and preoperative, intraoperative, and postoperative versions of the same model were retained as distinct model evaluations.

Predictive-Performance Data

The principal performance measures extracted were the area under the receiver operating characteristic curve (AUC) or concordance statistic, together with its 95% confidence interval, sensitivity, and specificity. Other measures, including accuracy, positive predictive value, negative predictive value, F1 score, calibration slope, calibration intercept, calibration plots, Brier score, and decision-curve measures, were also recorded when available. However, only measures reported sufficiently and consistently across the included studies were incorporated into the main comparative tables.

Where performance was reported for both model-development and validation datasets, estimates from an independent test set, temporal validation set, geographical validation set, or external-validation cohort were preferentially extracted for the principal presentation. Apparent performance calculated using the model-development data was identified separately and was not considered equivalent to validated performance. When multiple thresholds were reported, the threshold selected by the primary investigators for the principal model evaluation was extracted. Performance measures were not recalculated when the required participant-level data or two-by-two contingency tables were unavailable.

The term “not reported” was used when an eligible item or performance measure was not provided. Study-level characteristics were presented once for each study, whereas model-level performance was presented separately for every eligible algorithm to avoid conflating the number of studies with the number of models evaluated.

Risk of Bias and Applicability Assessment

The methodological quality, risk of bias, and applicability of the included prediction-model studies were evaluated using the PROBAST+AI tool [17]. PROBAST+AI was selected because it applies to prediction models developed using regression, machine-learning, or other AI methods and explicitly distinguishes the methodological quality of model development from the risk of bias in model-performance evaluation. The model-development component assesses the methodological quality and applicability of the development process, whereas the model-evaluation component assesses the risk of bias and applicability of the model-performance evaluation. Both components address four domains: participants and data sources, predictors, outcome, and analysis.

Applicability was assessed in relation to the first three domains: participants and data sources, predictors, and outcome. The development component contains 16 signaling questions, while the model-evaluation component contains 18 signaling questions.

For the participants and data-sources domain, reviewers considered whether the data source, eligibility criteria, exclusions, and participant selection were appropriate and representative of the population in which the model was intended to be used. The predictors domain assessed whether predictors were clearly defined, measured consistently, available at the intended time of prediction, and assessed without inappropriate knowledge of the outcome. The outcome domain considered the appropriateness, consistency, objectivity, and blinding of outcome definition and measurement and whether the prediction interval was clinically appropriate.

The analysis domain considered the adequacy of the sample size and number of outcome events, appropriate handling of continuous predictors and missing data, avoidance of inappropriate predictor selection, prevention of data leakage, management of class imbalance, separation of training and evaluation data, control of overfitting and optimism, appropriateness of internal or external validation, reporting of discrimination and calibration, and whether the performance estimates corresponded to the final model.

The unit of assessment was the individual model evaluation rather than the publication alone. Where several algorithms within a study used the same participants, predictors, outcome definition, preprocessing, and validation strategy, a common domain judgment was applied where justified, with algorithm-specific differences documented separately.

Two reviewers independently completed the PROBAST+AI assessments. Each signaling question was answered according to the tool guidance, and domain-level and overall judgments were categorized as low, high, or unclear concern or risk, as applicable. Disagreements were resolved through discussion or adjudication by a third reviewer. No numerical quality score was calculated. Risk-of-bias findings were summarized graphically and incorporated into the interpretation of model performance.

Data Synthesis

Narrative synthesis was undertaken because quantitative pooling was not considered methodologically appropriate. Synthesis was structured using the principles of the Synthesis Without Meta-analysis guideline, adapted for prediction-model evidence [18].

Studies were first grouped according to the clinical outcome predicted, namely PONV, perioperative hypotension, prolonged ICU stay, in-hospital mortality, and neurological complications. Study characteristics were summarized in a general characteristics table containing the country, design, participant characteristics, surgical specialty, mean or median age, sample size, predicted outcome, and risk-of-bias assessment. Separate outcome-specific tables were then constructed to present the type of surgery, algorithm, predictors or model parameters, AUC with its confidence interval, sensitivity, and specificity.

Within each outcome group, models were described according to model family, predictor domains, point of prediction, and validation approach. Models were also distinguished according to whether they used predominantly preoperative data, intraoperative physiological or medication data, postoperative data, or combinations of data collected across the perioperative pathway. When several models were evaluated within the same study population, all eligible models were presented, but they were recognized as statistically dependent evaluations rather than independent studies.

Meta-analysis was not performed because of substantial clinical and methodological heterogeneity across the included studies. Specifically, the studies differed in predicted outcomes and outcome definitions, outcome-incidence rates and prediction horizons, surgical specialties and patient case mix, timing and availability of predictors, number and type of candidate predictors, AI and machine-learning algorithms, preprocessing and feature-selection methods, approaches to missing data and class imbalance, model-development and tuning procedures, internal and external validation strategies, performance datasets, classification thresholds, and reported performance measures. Furthermore, several studies evaluated multiple algorithms using the same participant cohort, creating non-independent estimates, while many did not provide confidence intervals, calibration measures, common classification thresholds, or sufficient two-by-two data for reconstruction of comparable accuracy estimates. Pooling AUCs, sensitivities, or specificities under these circumstances could therefore produce a statistically precise but clinically uninterpretable summary.

For each outcome, narrative synthesis described the range and consistency of reported AUC, sensitivity, and specificity values; algorithms showing comparatively higher performance within the same study; the predictor domains used; and the extent of internal or external validation. Direct comparisons between algorithms were emphasized only when they had been evaluated in the same population using the same outcome definition and validation dataset. Differences in performance between unrelated studies were interpreted cautiously and were not treated as evidence that one algorithm was intrinsically superior to another. Greater interpretive weight was given to models evaluated using independent or external datasets and to studies with lower concerns regarding risk of bias and applicability. Findings from studies at high or unclear risk of bias were retained but interpreted cautiously.

Results

Study Selection

Database searches identified 2,329 records. After removing 477 duplicate records, 1,852 unique records underwent title and abstract screening, of which 1,682 were excluded. All 170 reports sought for full-text retrieval were obtained and assessed for eligibility. Of these, 148 were excluded: 77 did not involve the prespecified population undergoing general anesthesia, 52 did not evaluate an eligible prediction model, and 19 did not report relevant outcome or model-performance data. Overall, 22 studies were included in the systematic review (Figure 1) [5-8,10-13,19-32].

**Figure 1:**
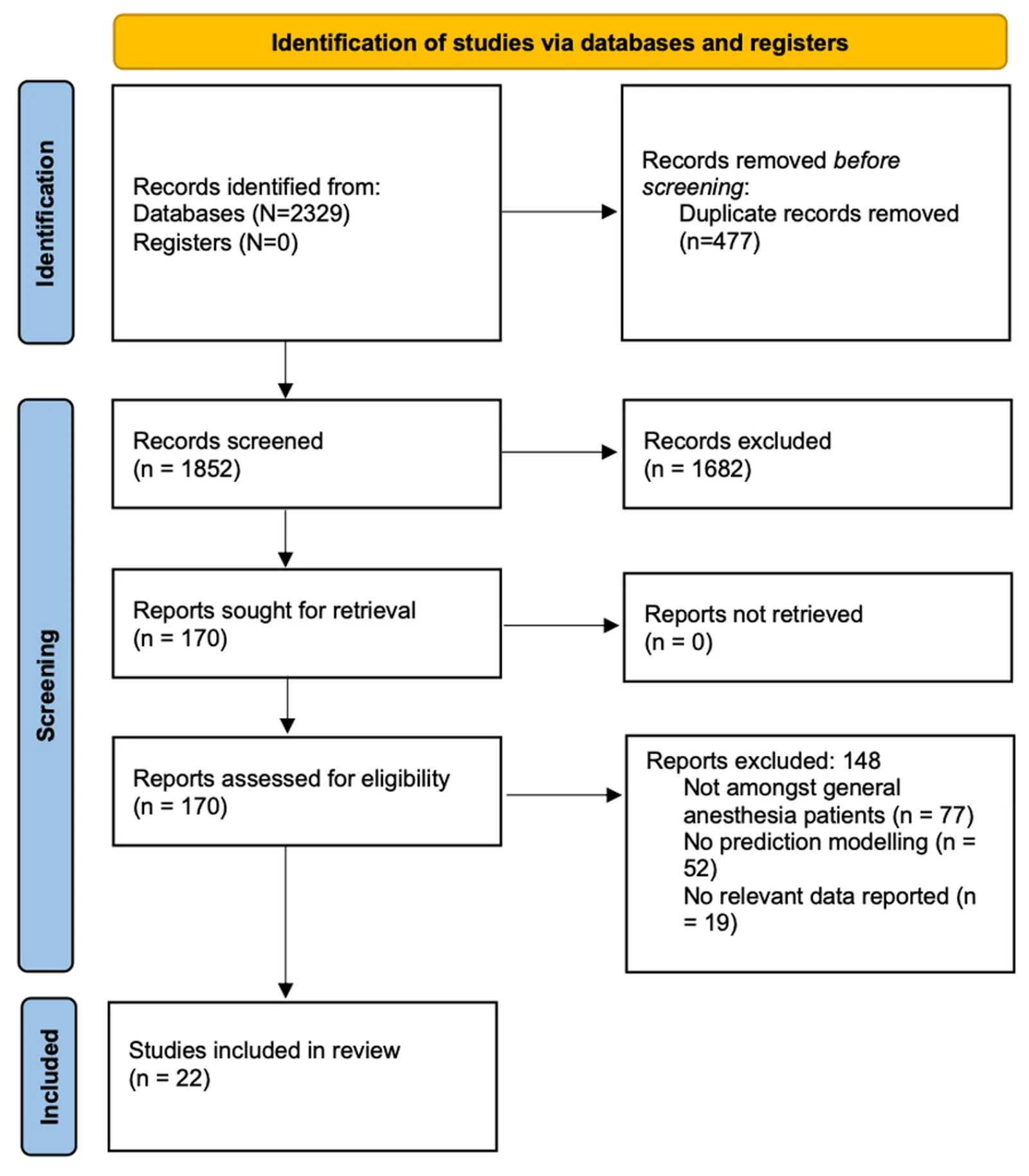
PRISMA flowchart PRISMA, Preferred Reporting Items for Systematic Reviews and Meta-Analyses

Characteristics of the Included Studies

Overall, 22 studies were published between 2003 and 2025 and reported data from 446,201 participants across study reports, with individual sample sizes ranging from 221 to 106,860 participants (Table 3). Fifteen studies evaluated PONV, five evaluated hypotension, and two evaluated prolonged ICU stay, in-hospital mortality, and neurological complications.

**Table 3:** Characteristics of the included studies (N=22). ENT, ear, nose, and throat; EU, European Union; LC, laparoscopic cholecystectomy; NR, not reported; PACU, post-anesthesia care unit; PONV, postoperative nausea and vomiting; RCT, randomized controlled trial

Study (year)	Country	Study design	Characteristics of participants	Surgical specialty/type of surgery	Mean age (years)	Sample size	Outcome
Choi et al. (2024) [19]	South Korea	Models from RCT datasets	Adult patients who received postoperative opioids and had an Apfel score of 3 or higher.	Breast-conserving operation, modified radical mastectomy, and breast reconstruction	53 (median)	1,390	PONV
Fan et al. (2025) [20]	China	Retrospective cohort study	Adult patients aged eighteen to seventy-five years without unexpected termination of the day surgery plan.	Day-case LC	NR	2,709	PONV
Glebov et al. (2025) [21]	Israel	Retrospective study	Adult inpatients admitted to the PACU after undergoing surgical procedures under general anesthesia. Patients undergoing cardiac, obstetric, or local anesthesia procedures were excluded.	Various surgical procedures	51.0 (median)	35,003	PONV
Hoshijima et al. (2024) [7]	Japan	Retrospective study	Adult patients aged 20 years or older who underwent surgery under general anesthesia. Patients with severe intraoperative or postoperative complications were excluded.	Digestive, vascular, transplant, oral, gynecology, urology, orthopedics, otorhinolaryngology, neurosurgery, ophthalmology, plastic, and dermatology	58.7	33,676	PONV
Kim et al. (2023) [6]	Republic of Korea	Retrospective study	Adult patients who underwent surgery under general anesthesia. Patients who received ventilator care after surgery or discharged within 24 hours were excluded.	Abdomen, breast, ear, endocrinologic, eye, head and neck, musculoskeletal, neurosurgery, obstetric, gynecologic, spine, thoracic, transplantation, urogenital, vascular, and skin surgeries	53.0 (median)	106,860	PONV
Lee et al. (2007) [23]	Korea	Prospective study	Patients using patient-controlled analgesia after their operations. Patients with preoperative use of narcotics, postoperative hypoxia, or transferred to the ICU were excluded.	Various surgeries including laparoscopic procedures	NR	1,181	PONV
Luo et al. (2024) [25]	China	Retrospective and multicenter study	Adult patients who underwent surgery under general anesthesia with patient-controlled intravenous analgesia. Patients suffering from gastrointestinal and central nervous system diseases were excluded.	Gynecologic laparoscopic surgery	48 to 49 (median)	1,233	PONV
Peng et al. (2007) [26]	Taiwan	Prospective study	Adult inpatients who underwent elective surgery under general anesthesia without antiemetic prophylaxis. Patients undergoing ophthalmic procedures or needing postoperative ventilator support were excluded.	Orthopedics, ENT, general, gynecological, urological, plastic, and neurosurgery	38 to 45	1,086	PONV
Shim et al. (2022) [5]	Republic of Korea	Retrospective study	Adult patients subjected to general anesthesia with intravenous patient-controlled analgesia. Patients subjected to regional anesthesia were excluded.	Abdominal, thoracic, obstetric, gynecological, urology, brain, spine, shoulder, hip, extremities, skin, and soft tissue surgeries	60 (median)	2,149	PONV
Stallings-Welden et al. (2018) [27]	USA	Prospective randomized study	Ambulatory surgical patients randomly assigned to receive either standard care or aromatherapy.	General, orthopedic, neurological, urinary, head, neck, and vascular surgeries	NR	221	PONV
Suhre et al. (2020) [28]	USA	Retrospective observational study	Patients undergoing general anesthesia. Patients with an American Society of Anesthesiologists Physical Classification score of 4 or greater were excluded.	Non-obstetric and noncardiac procedures	48 to 53	27,388	PONV
Traeger et al. (2003) [29]	Germany	Prospective observational study	Patients undergoing elective surgical procedures under balanced anesthesia.	Elective surgical procedures	NR	2,164	PONV
Xie et al. (2023) [30]	China	Retrospective study	Adult patients who underwent general anesthesia and postoperative patient-controlled analgesia. Patients admitted to the ICU after surgery were excluded.	Surgical procedures under general anesthesia	NR	2,758	PONV
Zhou et al. (2023) [32]	China	Retrospective study	Adult patients who underwent general anesthesia and surgery.	Gynecological, urological, or general surgical procedures	59.9 to 61.0	2,617	PONV
Zheng et al. (2024) [31]	China	Retrospective analysis	High-risk patients for PONV enrolled in a prospective trial.	Laparoscopic gastrointestinal surgery including gastrectomy, small intestinal resection, colon resection, and rectum resection	NR	1,154	PONV
Hatib et al. (2018) [8]	Multinational (USA, EU, Canada)	Retrospective and prospective observational study	Surgical patients more than 18 years old.	Operating room and ICU procedures	56.5 to 64.5	1888	Hypotension
Palla et al. (2022) [10]	USA	Mixed methods study	Surgical procedures performed on adults resulting in PACU stays. Cardiac, obstetric, non-operating theater, and procedures missing key features were excluded.	Orthopedics, general, urology, ENT, gynecology, ophthalmology, neurology, plastic, vascular, oral and maxillofacial, and thoracic surgeries	54 (Median)	104,875	Hypotension
Kendale et al. (2018) [22]	USA	Retrospective study	Patients over the age of 12 who underwent any procedure requiring general anesthesia.	Cardiovascular, gastroenterology, bariatrics, oral, robotic, oncology, transplant, wound care, gynecology, neurosurgical, obstetrics, ophthalmology, orthopedic, otolaryngology, plastic, pulmonary, thoracic, urology, and vascular surgeries	50	13,323	Hypotension
Li et al. (2021) [11]	China	Retrospective study	Patients 18 years of age or older who underwent cardiac surgery.	Cardiac surgery (coronary artery bypass graft and valve surgery)	62 to 63	3,030	Hypotension
Lin et al. (2011) [24]	Taiwan	Retrospective study	Patients who underwent general anesthesia.	General anesthesia	46.4 to 46.5	1,311	Hypotension
Datta et al. (2020) [12]	USA	Retrospective cohort analysis	Adult patients admitted to the hospital for longer than 24 hours following any type of operative procedure. Patients who died during surgery or had incomplete records were excluded.	Orthopedic, neurosurgery, vascular, thoracic/cardiovascular, urologic, trauma-acute care, gastrointestinal, ENT, gynecology obstetrics, pancreas and biliary, transplant, plastic, burn, pediatric, ophthalmology, and other surgeries	56.5 to 57.5	43,943	Prolonged ICU Stay, neurological complications, and in-hospital mortality
Shickel et al. (2023) [13]	USA	Retrospective, longitudinal cohort study	Patients admitted to the hospital who were at least 18 years of age and underwent at least one surgical procedure during hospitalization. Patients with intraoperative mortality or missing variables were excluded.	Orthopedic, neurosurgery, urologic, vascular, otolaryngology, cardiothoracic, gastrointestinal, gynecologic, oncology, plastic, burn, transplantation, and other surgeries	59.0 to 61.0 (median)	56,242	Prolonged ICU stay, neurological complications, and in-hospital mortality

Thirteen studies were conducted in East Asia, including six in China, four in South Korea, two in Taiwan, and one in Japan. Six were conducted in the USA, one in Israel, one in Germany, and one used multinational data from the USA, Europe, and Canada. The clinical populations ranged from broad mixed-surgical cohorts to narrowly defined groups undergoing breast surgery, day-case laparoscopic cholecystectomy, gynecological laparoscopy, gastrointestinal surgery, cardiac surgery, or procedures followed by intravenous patient-controlled analgesia. Models incorporated combinations of demographic characteristics, medical history, laboratory measurements, surgical and anesthetic characteristics, medication exposures, high-frequency physiological data, postoperative pain, and resource-use variables. Algorithms included artificial neural networks, multilayer perceptrons, recurrent neural networks, decision trees, random forests, support vector machines, k-nearest-neighbor methods, Bayesian classifiers, gradient-boosting frameworks, and ensemble models.

Risk of Bias and Applicability

Under the conservative study-level PROBAST+AI assessment, all 22 studies were judged to have high overall risk of bias in reported model-performance evaluation (Table 4). The analysis domain was the principal source of concern and was rated high risk in all studies. Recurrent limitations included incomplete evaluation of calibration, reliance on random split-sample or cross-validation procedures without independent geographical validation, unclear separation of feature selection and hyperparameter optimization from model testing, incomplete reporting of missing-data methods, threshold selection within the development data, and inadequate adjustment for optimism after evaluating multiple algorithms.

**Table 4:** PROBAST+AI assessment of risk of bias and applicability of the included prediction-model studies. High indicates a high risk of bias or high applicability concern; low indicates a low risk of bias or low applicability concern; Unclear indicates insufficient information for a definitive judgment. PONV, postoperative nausea and vomiting; ICU, intensive care unit; PACU, post-anesthesia care unit

Study	Participants and data sources	Predictors	Outcome	Analysis	Overall risk of bias	Applicability concern	Principal basis for judgment
Choi et al. (2024) [19]	High	Low	Low	High	High	High	Prediction dataset combined selected randomized trial cohorts of predominantly high-risk women undergoing breast surgery; PONV was assessed using a structured instrument, but model performance was not established through independent external validation and calibration was not reported.
Fan et al. (2025) [20]	Unclear	High	Unclear	High	High	High	Single-procedure, day-case cohort; postoperative pain and postoperative analgesic use were included as predictors, creating potential temporal overlap with PONV; extensive resampling and feature selection increased overfitting concerns.
Glebov et al. (2025) [21]	Low	Unclear	Unclear	High	High	Low	Large, broadly defined surgical cohort, but the timing and completeness of some physiological predictors and electronic-record ascertainment of early and delayed PONV were insufficiently clear; evaluation remained single-center and internally validated.
Hoshijima et al. (2024) [7]	High	Low	High	High	High	Low	Exclusion of patients with severe perioperative complications could have produced a selected cohort; PONV was identified from retrospective records and antiemetic administration; random data splitting did not address transportability or calibration.
Kim et al. (2023) [6]	High	Low	High	High	High	Low	Patients receiving postoperative ventilation and those discharged within 24 hours were excluded, potentially producing outcome-dependent selection; retrospective outcome capture and high-dimensional model selection introduced additional concerns.
Lee et al. (2007) [23]	High	High	Unclear	High	High	High	Restricted to patients receiving patient-controlled analgesia, with postbaseline exclusions; postoperative pain and commencement of oral intake were used as predictors during the period in which PONV could already have occurred; validation reporting was limited.
Luo et al. (2024) [25]	Low	Low	Unclear	High	High	High	Multicenter cohort with predictors generally available before outcome assessment, but PONV was retrospectively ascertained; handling of missingness, variable selection, model optimism, and independent validation was insufficiently reported.
Peng et al. (2007) [26]	Unclear	Low	Low	High	High	Low	Prospective outcome assessment and clearly timed predictors reduced concerns, but participant-selection details and contemporary methods for handling optimism, calibration, and external validation were limited.
Shim et al. (2022) [5]	High	Low	Unclear	High	High	High	Restricted to patients receiving fentanyl-based intravenous patient-controlled analgesia; retrospective 24-hour outcome ascertainment and internal testing only; substantial sensitivity–specificity imbalance indicated threshold and class-imbalance concerns.
Stallings-Welden et al. (2018) [27]	High	High	Low	High	High	High	Secondary prediction analysis of a small randomized study, with only 64 PONV events; several treatment or postoperative variables could have been measured after risk had evolved; multiple algorithms were evaluated without robust independent validation.
Suhre et al. (2020) [28]	Unclear	High	High	High	High	Low	Retrospective cohort with self-reported cannabis exposure and PACU opioid or antiemetic variables that might overlap temporally with the outcome; PONV ascertainment, calibration, and principal model-performance estimates were incompletely reported.
Traeger et al. (2003) [29]	Unclear	Unclear	Unclear	High	High	Unclear	Prospective design, but sparse reporting of participant selection, predictor measurement, outcome ascertainment, missing data, network development, and independent validation precluded confident low-risk judgments.
Xie et al. (2023) [30]	High	Low	Unclear	High	High	High	Restricted to patient-controlled analgesia recipients and excluded postoperative ICU admissions; although an external cohort was used, extensive oversampling, feature selection, and algorithm comparison increased optimism concerns, and calibration was not reported.
Zhou et al. (2023) [32]	Unclear	Low	Unclear	High	High	Unclear	Retrospective cohort with limited surgical categories; severe class imbalance was reflected by sensitivities of approximately 0.01-0.15 for several models; no independent external validation or calibration was reported.
Zheng et al. (2024) [31]	High	Unclear	Low	High	High	High	Secondary analysis of high-risk participants enrolled in a clinical trial; several prophylaxis- and treatment-related predictors had uncertain timing; modest sample size and internal model comparison without external validation increased overfitting concerns.
Hatib et al. (2018) [8]	High	Low	Low	High	High	High	Selected patients with high-fidelity arterial waveforms from operating-room and ICU settings; outcome was objectively defined and predictors preceded the event, but event-window sampling, repeated observations within individuals, proprietary development, and limited calibration information raised analytical concerns.
Palla et al. (2022) [10]	Low	Low	Low	High	High	Low	Large surgical cohort with routinely available predictors measured before an objectively defined PACU outcome; however, performance was evaluated within a single health system, and missing-data handling, calibration, and geographical validation were insufficiently established.
Kendale et al. (2018) [22]	Low	Low	Low	High	High	Low	Broad surgical cohort and objective postinduction hypotension definition; concerns centerd on complete-case selection, internal model selection and validation, absence of external validation, and limited calibration reporting.
Li et al. (2021) [11]	Low	Low	Low	High	High	High	Predictors and hypotension outcome were appropriately timed and objectively measured, but the model was developed in a single-center cardiac-surgery cohort with internal validation and no demonstrated external calibration or transportability.
Lin et al. (2011) [24]	Unclear	Low	Low	High	High	Unclear	Objective outcome and appropriately timed clinical predictors, but retrospective participant selection, missing-data methods, neural-network optimization, calibration, and independent validation were insufficiently reported.
Datta et al. (2020) [12]	Unclear	Low	Unclear	High	High	Low	Large electronic-health-record cohort, but exclusion of incomplete records could introduce selection; ICU stay and mortality were objective, whereas neurological complications represented a coded composite; evaluation remained within the development health system.
Shickel et al. (2023) [13]	Unclear	Low	Unclear	High	High	Low	Large longitudinal cohort with appropriately sequenced perioperative inputs; exclusions for missing data and composite neurological outcomes raised uncertainty, while numerous model and outcome evaluations were performed without independent health-system validation or comprehensive calibration reporting.

The domain related to participant information had a high risk of bias in nine studies and an unclear risk of bias in eight studies. Similar ratings were found for the domain related to data sources information. Concerns arose from selected trial populations, restriction to patient-controlled analgesia recipients or high-risk patients, exclusion of participants according to postoperative events, and inclusion only of patients with specialized monitoring data. The predictor domain was rated high risk in four studies, mainly when postoperative pain, analgesic administration, oral intake, PACU treatments, or other variables could have been recorded during the same period as outcome development. The outcome domain was rated high risk in 3 studies and unclear in 10 studies, primarily because retrospective PONV ascertainment depended on routine documentation or antiemetic administration rather than uniform prospective assessment.

Overall applicability concerns were high in 10 studies, predominantly because models were developed in procedure-specific cohorts, high-risk trial participants, patient-controlled analgesia populations, cardiac-surgery patients, or patients with invasive arterial waveform monitoring. Applicability was lower for the large mixed-surgical electronic-health-record cohorts, although geographical and institutional transportability remained uncertain.

Narrative Synthesis of Model Performance

Because the models differed substantially in their patient populations, outcome definitions, prediction horizons, predictor timing, algorithm development, validation procedures, and classification thresholds, no pooled performance estimate was calculated. AUC values were used to describe discrimination, whereas sensitivity and specificity were interpreted as threshold-dependent measures and were not compared directly across unrelated cohorts.

Postoperative Nausea and Vomiting

Fifteen studies, comprising 221,589 participants across reports, evaluated AI-based prediction of PONV (Table 5). Studies covered general surgical populations and selected cohorts undergoing breast, laparoscopic cholecystectomy, gynecological laparoscopic, gastrointestinal, and ambulatory procedures, as well as patients receiving intravenous patient-controlled analgesia. Reported AUCs ranged from 0.561 to 0.960. Sensitivity ranged from 0.010 to 1.000 and specificity from 0 to 0.992, demonstrating marked dependence on model threshold, outcome prevalence, and management of class imbalance. Confidence intervals and calibration measures were inconsistently reported.

**Table 5:** Characteristics and predictive performance of artificial intelligence models for PONV in patients undergoing general anesthesia. ANN, artificial neural network; ASA, American Society of Anesthesiologists; BART, Bayesian Additive Regression Trees; BMI, body mass index; BSMOTE, borderline synthetic minority oversampling technique; BOR, Boruta feature selection; CB, CatBoost; cforest, conditional random forest; CNNRNN, convolutional neural network–recurrent neural network; DT, decision tree; DNN, deep neural network; GBDT, gradient boosting decision tree; GBM, gradient boosting machine; KNN, k-nearest neighbor; LA, Least Absolute shrinkage and selection operator screening; LC, laparoscopic cholecystectomy; LESS, laparoendoscopic single-site surgery; LGBM, light gradient boosting machine; LightGBM, light gradient boosting machine; LSTM, long short-term memory; MLP, multilayer perceptron; NLR, neutrophil-to-lymphocyte ratio; NR, not reported; PACU, post-anesthesia care unit; PaO₂, partial pressure of arterial oxygen; PCA, patient-controlled analgesia; PETCO₂, end-tidal carbon dioxide partial pressure; PONV, postoperative nausea and vomiting; RF, random forest; RFE, recursive feature elimination; RNN, recurrent neural network; SAS, self-rating anxiety scale; SGD, stochastic gradient descent; SMOTE, synthetic minority oversampling technique; SMO, sequential minimal optimization; SVC, support vector classifier; SVM, support vector machine; XGBoost, extreme gradient boosting

Study	Type of surgery	Model name	Model parameters	Model AUC with CI	Sensitivity	Specificity
Choi et al. (2024) [19]	Breast surgery and other procedures	Classification tree	Female, history of PONV or motion sickness, nonsmoking, age, postoperative opioids, ASA physical status, BMI, method of administration of prophylactic antiemetic	0.584 (0.527-0.639)	0.606	0.535
Choi et al. (2024) [19]	Breast surgery and other procedures	Random forest	Female, history of PONV or motion sickness, nonsmoking, age, postoperative opioids, ASA physical status, BMI, method of administration of prophylactic antiemetic	0.591 (0.546-0.641)	0.536	0.583
Choi et al. (2024) [19]	Breast surgery and other procedures	XGBoost	Female, history of PONV or motion sickness, nonsmoking, age, postoperative opioids, ASA physical status, BMI, method of administration of prophylactic antiemetic	0.580 (0.528-0.630)	0.555	0.558
Choi et al. (2024) [19]	Breast surgery and other procedures	Neural network	Female, history of PONV or motion sickness, nonsmoking, age, postoperative opioids, ASA physical status, BMI, method of administration of prophylactic antiemetic	0.636 (0.593-0.684)	0.642	0.568
Fan et al. (2025) [20]	Day-case LC	KNN_BSMO_RFE_LGBM	Gender, age, history of allergies, history of hypertension, history of diabetes, preoperative creatinine, preoperative blood sugar, preoperative glomerular filtration rate, intraoperative use of sevoflurane, intraoperative sufentanil dosage, intraoperative dosage of sufentanil per kilogram of body weight, intraoperative dosage of cisatracurium, intraoperative dosage of cisatracurium per kilogram of body weight, intraoperative use of hydromorphone, intraoperative use of tropisetron, use of endotracheal tube, LESS, postoperative pain, postoperative dezocine use, postoperative lofencodeine use	0.96	0.87	0.954
Fan et al. (2025) [20]	Day-case LC	KNN_SMO_RFE_LGBM	Gender, age, history of allergies, history of hypertension, history of diabetes, preoperative creatinine, preoperative blood sugar, preoperative glomerular filtration rate, intraoperative use of sevoflurane, intraoperative sufentanil dosage, intraoperative dosage of sufentanil per kilogram of body weight, intraoperative dosage of cisatracurium, intraoperative dosage of cisatracurium per kilogram of body weight, intraoperative use of hydromorphone, intraoperative use of tropisetron, use of endotracheal tube, LESS, postoperative pain, postoperative dezocine use, postoperative lofencodeine use	0.9538	0.884	0.96
Fan et al. (2025) [20]	Day-case LC	KNN_BSMO_RFE_MLP	Gender, age, history of allergies, history of hypertension, history of diabetes, preoperative creatinine, preoperative blood sugar, preoperative glomerular filtration rate, intraoperative use of sevoflurane, intraoperative sufentanil dosage, intraoperative dosage of sufentanil per kilogram of body weight, intraoperative dosage of cisatracurium, intraoperative dosage of cisatracurium per kilogram of body weight, intraoperative use of hydromorphone, intraoperative use of tropisetron, use of endotracheal tube, LESS, postoperative pain, postoperative dezocine use, postoperative lofencodeine use	0.7848	0.542	0.904
Fan et al. (2025) [20]	Day-case LC	Simp_BSMO_BOR_LGBM	Gender, age, history of allergies, history of hypertension, history of diabetes, preoperative creatinine, preoperative blood sugar, preoperative glomerular filtration rate, intraoperative use of sevoflurane, intraoperative sufentanil dosage, intraoperative dosage of sufentanil per kilogram of body weight, intraoperative dosage of cisatracurium, intraoperative dosage of cisatracurium per kilogram of body weight, intraoperative use of hydromorphone, intraoperative use of tropisetron, use of endotracheal tube, LESS, postoperative pain, postoperative dezocine use, postoperative lofencodeine use	0.7305	0.3593	0.9918
Glebov et al. (2025) [21]	Surgical procedures under general anesthesia	XGBoost (early PONV)	Biometric, medical, procedural, and physiological variables	0.872	0.819	NR
Glebov et al. (2025) [21]	Surgical procedures under general anesthesia	Ensemble: KNN + random forest (delayed PONV)	Biometric, medical, procedural, and physiological variables	0.708	0.749	NR
Hoshijima et al. (2024) [7]	Surgery under general anesthesia	LightGBM	Patient-related factors, time-related factors, fluid-related factors, anesthetic agent-related factors, patient positioning, laparoscopic or non-laparoscopic surgery	0.77 (0.74 to 0.79)	0.57	NR
Hoshijima et al. (2024) [7]	Surgery under general anesthesia	KNN	Patient-related factors, time-related factors, fluid-related factors, anesthetic agent-related factors, patient positioning, laparoscopic or non-laparoscopic surgery	0.59 (0.56 to 0.63)	0.12	NR
Kim et al. (2023) [6]	Surgery under general anesthesia	Random forest	Demographic characteristics, medical history, surgical details, and medication administration records (103 features)	0.80 (0.80-0.81)	0.33	NR
Kim et al. (2023) [6]	Surgery under general anesthesia	Light gradient boosting machine	Demographic characteristics, medical history, surgical details, and medication administration records (103 features)	0.85 (0.85-0.86)	0.1	NR
Kim et al. (2023) [6]	Surgery under general anesthesia	Multilayer perceptron	Demographic characteristics, medical history, surgical details, and medication administration records (103 features)	0.69 (0.69-0.70)	0.59	NR
Kim et al. (2023) [6]	Surgery under general anesthesia	Balanced random forest	Demographic characteristics, medical history, surgical details, and medication administration records (103 features)	0.68 (0.67-0.68)	0.71	NR
Lee et al. (2007) [23]	Surgery under general anesthesia	DT (C5.0 algorithm)	Gender, obesity, anxiety, history of previous PONV, history of motion sickness, inhalation of nitrous oxide during operation, use of inhalational agents, starting oral fluid/food intake after operation, and post-operative pain	NR	NR	NR
Luo et al. (2024) [25]	Gynecologic laparoscopic surgery	Neural network	Dosage of remifentanil, dosage of sufentanil in the analgesic pump, dosage of propofol during the surgery, duration of surgery, use of neostigmine, use of steroids, maximum value of the PETCO_2_	0.834	0.578	0.838
Peng et al. (2007) [26]	Surgery under general anesthesia	ANN	Gender, type of surgery, ASA status, duration of anesthesia, smoking habit, history of previous PONV, and use of postoperative opioid	0.814 (0.774-0.850)	0.779	0.85
Peng et al. (2007) [26]	Surgery under general anesthesia	Naïve Bayesian classifier	Gender, type of surgery, ASA status, duration of anesthesia, smoking habit, history of previous PONV, and use of postoperative opioid	0.570 (0.522-0.617)	0.183	0.957
Shim et al. (2022) [5]	Non-cardiac surgery under general anesthesia	KNN	Age, sex, BMI, history of motion sickness, history of PONV, ASA physical status, hypertension, duration of anesthesia, premedication, preintubation opioid, laparoscopic surgery, type of surgery, background dose of fentanyl in PCA	0.597 (0.537-0.656)	0.87	0.2
Shim et al. (2022) [5]	Non-cardiac surgery under general anesthesia	DT	Age, sex, BMI, History of motion sickness, history of PONV, ASA physical status, hypertension, duration of anesthesia, premedication, preintubation opioid, laparoscopic surgery, type of surgery, background dose of fentanyl in PCA	0.561 (0.498-0.625)	0.86	0.17
Shim et al. (2022) [5]	Non-cardiac surgery under general anesthesia	RF	Age, sex, BMI, History of motion sickness, history of PONV, ASA physical status, hypertension, duration of anesthesia, premedication, preintubation opioid, laparoscopic surgery, type of surgery, background dose of fentanyl in PCA	0.610 (0.552-0.668)	0.88	0.22
Shim et al. (2022) [5]	Non-cardiac surgery under general anesthesia	GBM	Age, sex, BMI, history of motion sickness, history of PONV, ASA physical status, Hypertension, duration of anesthesia, premedication, preintubation opioid, laparoscopic surgery, type of surgery, background dose of fentanyl in PCA	0.580 (0.520-0.639)	0.86	0.19
Shim et al. (2022) [5]	Non-cardiac surgery under general anesthesia	SVM	Age, sex, BMI, history of motion sickness, history of PONV, ASA physical status, hypertension, duration of anesthesia, premedication, preintubation opioid, laparoscopic surgery, type of surgery, background dose of fentanyl in PCA	0.649 (0.592-0.707)	0.88	0.25
Shim et al. (2022) [5]	Non-cardiac surgery under general anesthesia	ANN	Age, sex, BMI, History of motion sickness, history of PONV, ASA physical status, hypertension, duration of anesthesia, premedication, preintubation opioid, laparoscopic surgery, type of surgery, background dose of fentanyl in PCA	0.686 (0.630-0.742)	0.92	0.24
Stallings-Welden et al. (2018) [27]	Ambulatory surgical patients	cforest	Gender, smoking, motion sickness, PONV, postoperative opioids, age, BMI, and intraoperative time	0.69	NR	NR
Suhre et al. (2020) [28]	Non-obstetric, noncardiac procedures	BART	Cannabis use pattern, age less than 50 years, ASA classification, exposure to nitrous oxide, exposure to a potent volatile anesthetic agent, surgical duration, female sex, history of PONV or motion sickness, absence of patient reported tobacco use, receipt of an opioid drug in the PACU, total number of prophylactic anti-emetic drugs given pre- or intraoperatively	NR	NR	NR
Traeger et al. (2003) [29]	Elective surgical procedures under balanced anesthesia	ANN	PONV in ≤1/3 of surgeries, PONV in 1/3–2/3 of surgeries, PONV in >2/3 of surgeries, duration of anesthesia	0.742 (0.697-0.785)	0.722	0.645
Xie et al. (2023) [30]	Surgical procedures under general anesthesia	SMOTE_BOR_MLP	Type of surgery, age, sex, height, weight, BMI, past medical history, history of surgery, laparoscopic surgery, operative duration, infusion volume, intraoperative urine volume, blood loss, antiemetics used during surgery, nonopioids used during surgery, remifentanil consumption, sufentanil consumption, ephedrine, dexmedetomidine, PCA regimen, history of motion sickness and/or PONV	0.5995	1	0
Xie et al. (2023) [30]	Surgical procedures under general anesthesia	BSMOTE_BOR_SGD	Type of surgery, age, sex, height, weight, BMI, past medical history, history of surgery, laparoscopic surgery, operative duration, infusion volume, intraoperative urine volume, blood loss, antiemetics used during surgery, nonopioids used during surgery, remifentanil consumption, sufentanil consumption, ephedrine, dexmedetomidine, PCA regimen, history of motion sickness and/or PONV	0.6501	0.7876	0.4326
Xie et al. (2023) [30]	Surgical procedures under general anesthesia	BSMOTE_LA_XGB	Type of surgery, age, sex, height, weight, BMI, past medical history, history of surgery, laparoscopic surgery, operative duration, infusion volume, intraoperative urine volume, blood loss, antiemetics used during surgery, nonopioids used during surgery, remifentanil consumption, sufentanil consumption, ephedrine, dexmedetomidine, PCA regimen, history of motion sickness and/or PONV	0.9444	0.8394	0.9197
Xie et al. (2023) [30]	Surgical procedures under general anesthesia	BSMOTE_LA_CB	Type of surgery, age, sex, height, weight, BMI, past medical history, history of surgery, laparoscopic surgery, operative duration, infusion volume, intraoperative urine volume, blood loss, antiemetics used during surgery, nonopioids used during surgery, remifentanil consumption, sufentanil consumption, ephedrine, dexmedetomidine, PCA regimen, history of motion sickness and/or PONV	0.9469	0.8108	0.9041
Zhou et al. (2023) [32]	Surgery under general anesthesia	DT	Haloperidol, sex, age, history of smoking, and history of PONV	0.707	0.039	NR
Zhou et al. (2023) [32]	Surgery under general anesthesia	SVC	Haloperidol, sex, age, history of smoking, and history of PONV	0.731	0.029	NR
Zhou et al. (2023) [32]	Surgery under general anesthesia	gnb	Haloperidol, sex, age, history of smoking, and history of PONV	0.667	0.137	NR
Zhou et al. (2023) [32]	Surgery under general anesthesia	knn	Haloperidol, sex, age, history of smoking, and history of PONV	0.623	0.088	NR
Zhou et al. (2023) [32]	Surgery under general anesthesia	adab	Haloperidol, sex, age, history of smoking, and history of PONV	0.722	0.078	NR
Zhou et al. (2023) [32]	Surgery under general anesthesia	DNN	Haloperidol, sex, age, history of smoking, and history of PONV	0.694	0.049	NR
Zhou et al. (2023) [32]	Surgery under general anesthesia	RNN	Haloperidol, sex, age, history of smoking, and history of PONV	0.611	0.147	NR
Zhou et al. (2023) [32]	Surgery under general anesthesia	LSTM	Haloperidol, sex, age, history of smoking, and history of PONV	0.672	NR	NR
Zhou et al. (2023) [32]	Surgery under general anesthesia	CNNRNN	Haloperidol, sex, age, history of smoking, and history of PONV	0.668	0.01	NR
Zheng et al. (2024) [31]	Laparoscopic gastrointestinal surgery	RF	BMI, Apfel score, preoperative SAS score, plasma fibrinogen, NLR, addition of fosaprepitant for PONV prophylactic therapy, nasogastric tube, cephalosporins, penicillins, nitroimidazoles, quinolones, pH, PaO_2_, blood potassium	0.737 (0.663, 0.812)	0.804	0.615
Zheng et al. (2024) [31]	Laparoscopic gastrointestinal surgery	XGBoost	BMI, Apfel score, preoperative SAS score, plasma fibrinogen, NLR, addition of fosaprepitant for PONV prophylactic therapy, nasogastric tube, cephalosporins, penicillins, nitroimidazoles, quinolones, pH, PaO_2_, blood potassium	0.737 (0.661, 0.813)	0.826	0.595
Zheng et al. (2024) [31]	Laparoscopic gastrointestinal surgery	KNN	BMI, Apfel score, preoperative SAS score, plasma fibrinogen, NLR, addition of fosaprepitant for PONV prophylactic therapy, nasogastric tube, cephalosporins, penicillins, nitroimidazoles, quinolones, pH, PaO_2_, blood potassium	0.695 (0.614,0.776)	0.696	0.581
Zheng et al. (2024) [31]	Laparoscopic gastrointestinal surgery	GBDT	BMI, Apfel score, preoperative SAS score, plasma fibrinogen, NLR, addition of fosaprepitant for PONV prophylactic therapy, nasogastric tube, cephalosporins, penicillins, nitroimidazoles, quinolones, pH, PaO_2_, blood potassium	0.730 (0.653,0.806)	0.848	0.518
Zheng et al. (2024) [31]	Laparoscopic gastrointestinal surgery	MLP	BMI, Apfel score, preoperative SAS score, plasma fibrinogen, NLR, addition of fosaprepitant for PONV prophylactic therapy, nasogastric tube, cephalosporins, penicillins, nitroimidazoles, quinolones, pH, PaO_2_, blood potassium	0.733 (0.656, 0.811)	0.674	0.718

In Choi et al.’s study, all four machine-learning models showed modest discrimination. The neural network performed best, with an AUC of 0.636, sensitivity of 0.642, and specificity of 0.568, whereas the classification tree, random forest, and XGBoost models had AUCs between 0.580 and 0.591 [19]. These models were derived from a selected population of women at increased PONV risk undergoing breast surgery, limiting the applicability beyond this setting.

In patients undergoing day-case laparoscopic cholecystectomy, Fan et al. reported the highest internal discrimination among the PONV studies [20]. Two LightGBM-based pipelines had AUCs of 0.960 and 0.954, with sensitivities of 0.870-0.884 and specificities of 0.954-0.960. However, performance varied substantially across alternative pipelines using the same predictors: another LightGBM configuration had an AUC of 0.731, sensitivity of 0.359, and specificity of 0.992. The inclusion of postoperative pain and postoperative analgesic administration also meant that these models did not represent purely preoperative risk prediction.

Glebov et al. distinguished early PONV from delayed PONV [21]. The XGBoost model for early PONV had an AUC of 0.872 and sensitivity of 0.819, whereas the ensemble model for delayed PONV had an AUC of 0.708 and sensitivity of 0.749. This difference suggests that predictability varied according to the postoperative assessment period rather than representing a single stable PONV phenotype.

In large mixed-surgical cohorts, Hoshijima et al. reported better discrimination with LightGBM than with k-nearest neighbors (AUC 0.770 vs 0.590) [7]. Kim et al. similarly found that LightGBM produced the highest AUC among the evaluated algorithms (0.850), compared with 0.800 for random forest, 0.690 for multilayer perceptron, and 0.680 for balanced random forest [6]. However, the LightGBM model had a sensitivity of only 0.10 at the selected threshold, whereas balanced random forest had lower discrimination but higher sensitivity of 0.71. Thus, higher AUC did not necessarily correspond to clinically acceptable identification of patients who developed PONV.

For gynecological laparoscopic surgery, the Luo neural-network model had an AUC of 0.834, sensitivity of 0.578, and specificity of 0.838 [25]. In the prospective study by Peng et al., the artificial neural network had an AUC of 0.814, sensitivity of 0.779, and specificity of 0.850, substantially outperforming the naïve Bayesian classifier within the same cohort, which had an AUC of 0.570 and sensitivity of 0.183 [26].

Among patients receiving fentanyl-based intravenous patient-controlled analgesia, the artificial neural network in Shim et al.’s study had the highest discrimination of the six evaluated machine-learning algorithms, although performance remained moderate (AUC 0.686). Sensitivities were high across these models, ranging from 0.86 to 0.92, but specificities were only 0.17-0.25, indicating a large number of false-positive classifications [5].

Xie et al. reported strong internal performance for models combining borderline synthetic minority oversampling with XGBoost or CatBoost, with AUCs of 0.944 and 0.947, respectively [30]. The CatBoost model had a sensitivity of 0.811 and a specificity of 0.904. By contrast, an alternative multilayer-perceptron model classified every participant as positive, producing a sensitivity of 1.00 and specificity of 0. The best model was subsequently evaluated in an external hospital cohort, where its AUC decreased from 0.9469 to 0.8211.

Zhou et al.’s study reported AUCs between 0.611 and 0.731 across conventional machine-learning and deep-learning algorithms. Despite apparently moderate discrimination for some models, sensitivities were extremely low, ranging from 0.010 to 0.147 for the reported thresholds [31]. In Zheng et al.’s study of high-risk gastrointestinal surgery patients, performance was more consistent across algorithms, with AUCs of 0.695-0.737. The random forest and XGBoost models had the highest AUCs, but the gradient-boosting decision tree had the highest sensitivity and the multilayer perceptron had the highest specificity [32]. Collectively, the PONV studies showed that model ranking depended on whether discrimination, sensitivity, or specificity was prioritized and that performance from internally optimized models did not establish generalizability.

Perioperative Hypotension

Five studies, comprising 124,427 participants across reports, evaluated hypotension prediction (Table 6). Target conditions were not uniform: they included impending intraoperative hypotension, postinduction hypotension, and hypotension in the PACU. AUCs ranged from 0.630 to 0.950, sensitivity from 57.0% to 92.0%, and specificity from 31.0% to 92.0%.

**Table 6:** Characteristics and predictive performance of artificial intelligence models for hypotension in patients undergoing general anesthesia. ANN, artificial neural network; AUC, area under the receiver operating characteristic curve; CABG, coronary artery bypass grafting; CI, confidence interval; HPI, hypotension prediction index; LightGBM, light gradient boosting machine; RF, random forest

Study	Type of surgery	Model name	Model parameters	Model AUC with CI	Sensitivity	Specificity
Hatib et al. (2018) [8]	Operating room and intensive care unit patients	Machine-learning algorithm (HPI) at 5 min	Individual features calculated from the high-fidelity arterial pressure waveform	0.95 (0.933-0.961)	92%	92%
Hatib et al. (2018) [8]	Operating room and intensive care unit patients	Machine-learning algorithm (HPI) at 10 min	Individual features calculated from the high-fidelity arterial pressure waveform	0.92 (0.896-0.943)	89%	90%
Hatib et al. (2018) [8]	Operating room and intensive care unit patients	Machine-learning algorithm (HPI) at 15 min	Individual features calculated from the high-fidelity arterial pressure waveform	0.91 (0.885-0.944)	88%	87%
Palla et al. (2022) [10]	Various (excluding cardiac and obstetric)	LightGBM	Preoperative and intraoperative data (routinely captured clinical variables from the electronic health record)	0.82 (0.812-0.832)	61.60%	83.80%
Kendale et al. (2018) [22]	Various (cardiovascular, gastroenterology, bariatrics, general, gynecology, neurosurgical, orthopedic, otolaryngology, plastic, thoracic, urology, vascular)	Support vector machines	Preoperative medications, medical comorbidities, induction medications, and intraoperative vital signs	0.59 (0.58-0.60)	84%	31%
Kendale et al. (2018) [22]	Various	Naïve Bayes	Preoperative medications, medical comorbidities, induction medications, and intraoperative vital signs	0.69 (0.67-0.69)	60%	70%
Kendale et al. (2018) [22]	Various	K-nearest neighbor	Preoperative medications, medical comorbidities, induction medications, and intraoperative vital signs	0.64 (0.63-0.65)	57%	68%
Kendale et al. (2018) [22]	Various	Linear discriminant analysis	Preoperative medications, medical comorbidities, induction medications, and intraoperative vital signs	0.72 (0.71-0.73)	70%	64%
Kendale et al. (2018) [22]	Various	RF	Preoperative medications, medical comorbidities, induction medications, and intraoperative vital signs	0.74 (0.73-0.75)	70%	65%
Kendale et al. (2018) [22]	Various	Neural nets	Preoperative medications, medical comorbidities, induction medications, and intraoperative vital signs	0.71 (0.69-0.71)	70%	63%
Kendale et al. (2018) [22]	Various	Stochastic gradient boosting machine	Preoperative medications, medical comorbidities, induction medications, and intraoperative vital signs	0.76 (0.75-0.77)	64%	75%
Li et al. (2021) [11]	Cardiac surgery (CABG, Valve surgery)	RF	Preoperative and perioperative variables	0.843 (0.808-0.877)	78.80%	85.60%
Lin et al. (2011) [24]	General anesthesia	ANN	Patient-related, 2 surgical, and 5 anesthetic variables (1 input layer with 18 input nodes and 1 output layer with 1 output node)	0.893	76.40%	85.60%

The waveform-based hypotension prediction index (HPI) evaluated by Hatib et al. had the highest reported discrimination. At prediction horizons of 5, 10, and 15 minutes, AUCs were 0.950, 0.920, and 0.910, respectively. Sensitivity and specificity declined as the prediction horizon increased, from 92% and 92% at 5 minutes to 88% and 87% at 15 minutes. These results indicated strong near-term discrimination but applied only to patients with sufficiently high-quality arterial pressure waveforms [8].

For prediction of PACU hypotension at the end of surgery, the LightGBM model evaluated by Palla et al. had an AUC of 0.820, sensitivity of 61.6%, and specificity of 83.8% [10]. In Kendale et al.’s study of postinduction hypotension, stochastic gradient boosting had the highest AUC among seven algorithms (0.760), with sensitivity of 64% and specificity of 75%. The support vector machine had an AUC of 0.630 and high sensitivity of 84% but specificity of only 31%, again illustrating the importance of the selected classification threshold [22].

Cardiac-surgery random-forest model developed by Li et al. had an AUC of 0.843, sensitivity of 78.8%, and specificity of 85.6% [11]. Lin et al. reported an AUC of 0.893, sensitivity of 76.4%, and specificity of 85.6% for an artificial neural network developed in a general anesthesia cohort. Direct ranking of these models against the HPI was not appropriate because the predicted events, available predictors, monitoring requirements, and prediction horizons differed [24].

Prolonged Intensive Care Unit Stay

Two studies evaluated prolonged ICU stay (Table 7). Datta et al. reported an AUC of 0.870 for a model using preoperative features and 0.880 after adding intraoperative and physiological data. The preoperative model had greater sensitivity (0.82 vs 0.75), whereas the perioperative model had greater specificity (0.87 vs 0.74). Thus, the small increase in discrimination was accompanied by a shift in the sensitivity-specificity balance rather than uniform improvement in all performance measures [12].

**Table 7:** Characteristics and predictive performance of artificial intelligence models for prolonged ICU stay in patients undergoing general anesthesia. AUC, area under the receiver operating characteristic curve; CI, confidence interval; ICU, intensive care unit; Intraop, intraoperative; Postop, postoperative; Preop, preoperative

Study	Type of surgery	Model name	Model parameters	Model AUC with CI	Sensitivity	Specificity
Datta et al. (2020) [12]	Inpatient surgeries	Random forest (preop model)	Preop features (demographic, socioeconomic, comorbidity, medication, laboratory value)	0.87 (0.86-0.87)	0.82 (0.81-0.83)	0.74 (0.74-0.75)
Datta et al. (2020) [12]	Inpatient surgeries	Random forest (postop model)	Demographic, socioeconomic, comorbidity, medication, laboratory value, operative, and physiological variables (134 preop and 233 intraop features)	0.88 (0.88-0.89)	0.75 (0.73-0.76)	0.87 (0.86-0.87)
Shickel et al. (2023) [13]	Major, inpatient surgery	Baseline preop (random forest)	Preop features, including demographic and socioeconomic indicators, planned procedure and provider information, Charlson comorbidities, and summary statistics of select medications, laboratory tests, and physiological measurements	0.87 (0.87-0.88)	0.81 (0.77-0.83)	0.77 (0.75-0.81)
Shickel et al. (2023) [13]	Major, inpatient surgery	Deep preop (individual)	Preop features	0.89 (0.88-0.89)	0.80 (0.78-0.83)	0.80 (0.77-0.82)
Shickel et al. (2023) [13]	Major, inpatient surgery	Baseline intraop (random forest)	Summary statistics extracted from each encounter’s intraop measurements (from 14 physiological measurements)	0.88 (0.87-0.88)	0.79 (0.77-0.83)	0.80 (0.76-0.82)
Shickel et al. (2023) [13]	Major, inpatient surgery	Deep intraop (individual)	Physiological measurements taken during surgery (variable-length multivariate time series)	0.89 (0.88-0.89)	0.81 (0.79-0.83)	0.82 (0.81-0.84)
Shickel et al. (2023) [13]	Major, inpatient surgery	Baseline postop (random forest)	Preop features, 49 summary statistics extracted from each encounter’s intraop measurements, blood loss sum, urine output sum, and duration of surgery	0.90 (0.89-0.90)	0.80 (0.78-0.82)	0.83 (0.81-0.86)
Shickel et al. (2023) [13]	Major, inpatient surgery	Deep postop (individual)	Preop features, 14 physiological measurements taken during surgery (variable-length multivariate time series), blood loss sum, urine output sum, and duration of surgery	0.92 (0.91-0.92)	0.83 (0.80-0.85)	0.84 (0.83-0.87)

In Shickel et al.’s study, AUCs ranged from 0.870 for the baseline preoperative random forest to 0.920 for the deep model incorporating information available by the end of surgery. Deep models had higher AUCs than the corresponding random-forest baselines for preoperative, intraoperative, and end-of-surgery predictions. The best-performing model had sensitivity of 0.83 and specificity of 0.84. These findings suggested incremental predictive value from integrating longitudinal intraoperative physiology with baseline information, although the estimates were generated within one institutional data environment [13].

In-Hospital Mortality

For in-hospital mortality, Datta et al. reported an increase in AUC from 0.870 with preoperative data to 0.930 after inclusion of intraoperative and physiological information (Table 8). Sensitivity increased from 0.83 to 0.85 and specificity from approximately 0.76 to 0.88, representing the largest incremental gain from intraoperative data among the three outcomes assessed in that study [12].

**Table 8:** Characteristics and predictive performance of artificial intelligence models for in-hospital mortality in patients undergoing general anesthesia. AUC, area under the receiver operating characteristic curve; CI, confidence interval; Intraop, intraoperative; Postop, postoperative; Preop, preoperative

Study	Type of surgery	Model name	Model parameters	Model AUC with CI	Sensitivity	Specificity
Datta et al. (2020) [12]	Inpatient surgeries	Random forest (preop model)	Preop features (demographic, socioeconomic, comorbidity, medication, laboratory value)	0.87 (0.84-0.90)	0.83 (0.73-0.87)	0.79 (0.78-0.80)
Datta et al. (2020) [12]	Inpatient surgeries	Random forest (postop model)	Demographic, socioeconomic, comorbidity, medication, laboratory value, operative, and physiological variables (134 preop and 233 intraop features)	0.93 (0.91-0.95)	0.85 (0.80-0.91)	0.88 (0.86-0.88)
Shickel et al. (2023) [13]	Major, inpatient surgery	Baseline preop (random forest)	Preop features, including demographic and socioeconomic indicators, planned procedure and provider information, Charlson comorbidities, and summary statistics of select medications, laboratory tests, and physiological measurements	0.84 (0.82-0.86)	0.75 (0.70-0.80)	0.82 (0.82-0.83)
Shickel et al. (2023) [13]	Major, inpatient surgery	Deep preop (individual)	Preop features	0.90 (0.88-0.91)	0.83 (0.80-0.92)	0.81 (0.72-0.81)
Shickel et al. (2023) [13]	Major, inpatient surgery	Baseline intraop (random forest)	Summary statistics extracted from each encounter’s intraop measurements (from 14 physiological measurements)	0.79 (0.77-0.82)	0.74 (0.64-0.79)	0.72 (0.72-0.84)
Shickel et al. (2023) [13]	Major, inpatient surgery	Deep intraop (individual)	Physiological measurements taken during surgery (variable-length multivariate time series)	0.86 (0.84-0.88)	0.85 (0.73-0.88)	0.72 (0.71-0.85)
Shickel et al. (2023) [13]	Major, inpatient surgery	Baseline postop (random forest)	Preop features, summary statistics extracted from each encounter’s intraop measurements, blood loss sum, urine output sum, and duration of surgery	0.83 (0.81-0.86)	0.72 (0.62-0.79)	0.82 (0.81-0.89)
Shickel et al. (2023) [13]	Major, inpatient surgery	Deep postop (individual)	Preop features, 14 physiological measurements taken during surgery (variable-length multivariate time series), blood loss sum, urine output sum, and duration of surgery	0.90 (0.89-0.92)	0.91 (0.78-0.94)	0.73 (0.72-0.85)

In Shickel et al.’s study, AUCs ranged from 0.790 for the baseline intraoperative random forest to 0.900 for both the deep preoperative and deep end-of-surgery models. The deep end-of-surgery model had the highest sensitivity of 0.91 but a specificity of 0.73. In contrast, the baseline preoperative model had lower sensitivity of 0.75 but specificity of 0.82. Consequently, the model with the highest sensitivity was not the model with the most balanced classification performance [13].

Neurological Complications

Datta et al. reported AUCs of 0.860 for the preoperative model and 0.890 after adding intraoperative data for neurological complications (Table 9). Sensitivity and specificity increased from 0.79 and 0.78 to 0.81 and 0.81, respectively [12].

**Table 9:** Characteristics and predictive performance of artificial intelligence models for neurological complications in patients undergoing general anesthesia. AUC, area under the receiver operating characteristic curve; CI, confidence interval; Intraop, intraoperative; Postop, postoperative; Preop, preoperative

Study	Type of surgery	Model name	Model parameters	Model AUC with CI	Sensitivity	Specificity
Datta et al. (2020) [12]	Inpatient surgeries	Random forest (preop model)	Preop features (demographic, socioeconomic, comorbidity, medication, laboratory value)	0.86 (0.85-0.87)	0.79 (0.77-0.80)	0.78 (0.77-0.78)
Datta et al. (2020) [12]	Inpatient surgeries	Random forest (postop model)	Demographic, socioeconomic, comorbidity, medication, laboratory value, operative, and physiological variables (134 preop and 233 intraop features)	0.89 (0.88-0.89)	0.81 (0.80-0.82)	0.81 (0.80-0.82)
Shickel et al. (2023) [13]	Major, inpatient surgery	Baseline preop (random forest)	Preop features, including demographic and socioeconomic indicators, planned procedure and provider information, Charlson comorbidities, and summary statistics of select medications, laboratory tests, and physiological measurements	0.83 (0.82-0.83)	0.79 (0.73-0.82)	0.70 (0.67-0.76)
Shickel et al. (2023) [13]	Major, inpatient surgery	Deep preop (individual)	Preop features	0.85 (0.84-0.86)	0.77 (0.73-0.83)	0.77 (0.71-0.81)
Shickel et al. (2023) [13]	Major, inpatient surgery	Baseline intraop (random forest)	Summary statistics extracted from each encounter’s intraop measurements (from 14 physiological measurements)	0.75 (0.74-0.76)	0.76 (0.68-0.77)	0.61 (0.60-0.69)
Shickel et al. (2023) [13]	Major, inpatient surgery	Deep intraop (individual)	Physiological measurements taken during surgery (variable-length multivariate time series)	0.75 (0.74-0.76)	0.70 (0.65-0.73)	0.67 (0.65-0.72)
Shickel et al. (2023) [13]	Major, inpatient surgery	Baseline postop (random forest)	Preop features, summary statistics extracted from each encounter’s intraop measurements, blood loss sum, urine output sum, and duration of surgery	0.81 (0.80-0.81)	0.76 (0.70-0.82)	0.70 (0.64-0.77)
Shickel et al. (2023) [13]	Major, inpatient surgery	Deep postop (individual)	Preop features, physiological measurements taken during surgery (variable-length multivariate time series), blood loss sum, urine output sum, and duration of surgery	0.86 (0.85-0.86)	0.80 (0.75-0.82)	0.75 (0.73-0.80)

In Shickel et al.’s study, neurological-complication AUCs ranged from 0.750 for models using intraoperative measurements alone to 0.860 for the deep end-of-surgery model. Preoperative models had AUCs of 0.830-0.850, suggesting that intraoperative physiology alone did not outperform information already available preoperatively. Performance improved when preoperative and intraoperative information was combined, with the deep end-of-surgery model attaining sensitivity of 0.80 and specificity of 0.75 [13].

Overall synthesis

AI models showed potentially useful discrimination for several perioperative outcomes, particularly near-term hypotension, prolonged ICU stay, mortality, and selected procedure-specific PONV models. Nevertheless, model performance was heterogeneous, and apparently high AUCs were not consistently accompanied by clinically acceptable sensitivity and specificity. Estimates were frequently derived from internally optimized models, calibration was incompletely reported, and independent external validation was uncommon. Consequently, the results support the technical feasibility of AI-based perioperative prediction but do not establish that any model is sufficiently validated for routine use across surgical populations and healthcare systems.

Discussion

This systematic review synthesised evidence from 22 studies evaluating AI-based prediction of PONV, perioperative hypotension, prolonged ICU stay, in-hospital mortality, and neurological complications among patients undergoing general anesthesia. Across outcomes, several models showed good or apparently excellent discrimination; however, performance was inconsistent across studies and was not always accompanied by an acceptable balance between sensitivity and specificity. Calibration, clinical utility, and external validation were reported much less frequently than discrimination. PROBAST+AI assessment consequently indicated substantial methodological concerns, particularly in the analysis domain, arising from internal validation alone, high-dimensional modelling in relatively restricted datasets, incomplete handling of missing data and class imbalance, possible temporal overlap between predictors and outcomes, and selective emphasis on the best-performing algorithm. These findings indicate that AI-based perioperative prediction is technically promising but remains predominantly at the model-development and internal-evaluation stage rather than at the stage of dependable clinical implementation.

The overall findings are consistent with earlier systematic reviews of machine learning in perioperative medicine. Bellini et al. reported that machine-learning applications frequently showed promising predictive performance but were heterogeneous in design, outcome selection, and validation, limiting direct comparison and clinical translation [33]. A larger review by Arina et al. subsequently identified 103 perioperative prediction studies and found that most evaluations were conducted within a single center, only a small minority underwent multicenter external validation, and the overall risk of bias was high [34]. The present review reaches a similar conclusion but provides a more clinically focused synthesis restricted to patients undergoing general anesthesia and to five outcomes that range from frequent patient-centered complications to major physiological and prognostic events. It additionally demonstrates that the principal limitation is not simply variability in AUC: threshold-dependent sensitivity and specificity can differ substantially even among algorithms evaluated in the same cohort, and strong discrimination may coexist with clinically unacceptable false-positive or false-negative rates.

For PONV, findings extend the established literature on conventional risk stratification. The simplified Apfel score remains attractive because it uses a small number of variables available before or during anesthesia and links risk categories to prophylactic decision-making [2-4]. AI models can incorporate substantially more information, including laboratory measurements, precise medication doses, surgical characteristics, postoperative pain, physiological measurements, and prophylaxis. Nevertheless, the present review found no consistent evidence that increasing model complexity invariably produced more clinically useful predictions. In some cohorts, neural networks or gradient-boosting methods performed better than simpler alternatives, whereas in others, their improvement was modest or accompanied by poor sensitivity. This finding is compatible with the broader prediction literature, in which flexible algorithms can model nonlinear relationships but cannot compensate for weak outcome ascertainment, limited event numbers, treatment-related predictors, or a lack of transportability. Contemporary perioperative risk-modelling reviews similarly emphasize that complex machine-learning approaches require demonstration of calibration, external validity, usability, and clinical value rather than reliance on discrimination alone [35].

The hypotension findings agree with the systematic review and meta-analysis by Mohammadi et al., which found strong discrimination for the HPI and somewhat lower pooled performance for non-HPI machine-learning models [36]. That review also reported evidence that HPI-guided care could reduce several measures of hypotension exposure, although publication bias and differences in study design remained concerns. The present synthesis reinforces the strong near-term predictive capability of arterial waveform analysis but also highlights its restricted applicability: these systems require an invasive arterial signal of sufficient quality and predict an event that may be only minutes away. Prediction performance alone therefore cannot determine whether use of the system improves outcomes, as benefit depends on alarm timing, clinician response, treatment protocol, hemodynamic phenotype, and the adverse consequences of unnecessary intervention.

Evidence from clinical-effectiveness reviews is more cautious than evidence from model-development studies. Shimada et al. found that randomized trials of AI-assisted anesthetic management were few, generally small, and largely centered on HPI-based management [37]. Mehta et al. found that HPI-supported interventions reduced measures of intraoperative hypotension, but evidence of improvement in broader patient-centered outcomes or length of stay was limited [38]. This gap between predictive performance and clinical effectiveness is central to interpreting the present review. An algorithm may accurately forecast an event without changing care, it may change care without improving outcomes, or it may reduce an intermediate physiological endpoint without preventing organ injury, disability, or death. The current evidence therefore supports a distinction between model accuracy, clinical decision support, and demonstrated patient benefit.

For ICU stay, mortality, and neurological complications, the present findings are also broadly aligned with previous perioperative reviews that identified frequent improvements after combining preoperative and intraoperative data but noted limited external validation and implementation evidence [33,34]. The relatively favorable discrimination observed in large electronic health record cohorts probably reflects their ability to capture numerous dimensions of patient vulnerability and operative stress. However, previous reviews have cautioned that models derived from a single health system may encode institution-specific documentation, case selection, postoperative pathways, and resource-allocation practices [34,35]. This concern is particularly relevant to ICU-related outcomes and composite complications. A model that performs well in the institution in which its outcome definitions and care processes were created may require substantial recalibration or redevelopment before use elsewhere.

Several clinical and methodological mechanisms may explain why PONV model performance varied so widely. PONV is a multifactorial outcome arising from interactions among patient susceptibility, surgical stimulation, anesthetic exposure, opioid use, prophylaxis, postoperative pain, and recovery processes. A model based solely on preoperative data addresses baseline susceptibility, whereas a model containing intraoperative medications or postoperative analgesia estimates risk conditional on treatments and events that have already occurred. Including such dynamic information can improve statistical prediction, but it may reduce the time available for prevention. Treatment variables also create a form of the treatment paradox: patients judged to be at high risk are more likely to receive prophylaxis, and successful prophylaxis may make the treated high-risk patient appear similar to a low-risk patient. Unless treatment selection is explicitly accounted for, the algorithm may learn local prescribing patterns rather than untreated biological risk.

Class imbalance provides another explanation for the marked divergence between PONV AUC, sensitivity, and specificity. When an outcome is relatively uncommon in the analyzed dataset, an algorithm can achieve apparently reasonable overall discrimination while performing poorly at the threshold used to identify cases. Oversampling and synthetic minority techniques can help models learn patterns among patients with the outcome, but they can also increase optimism if applied before data splitting or if evaluation is performed in a sample whose event distribution differs from clinical practice. The exceptionally high performance of some resampling-based pipelines, contrasted with poor performance of alternative pipelines in the same dataset and reduced discrimination in external validation, is consistent with this concern. Model selection based on the best result among many preprocessing and algorithm combinations further increases the risk that the reported estimate captures dataset-specific noise.

The comparatively strong short-horizon prediction of hypotension has a physiological explanation. Arterial pressure waveforms contain subtle changes in pulse contour, contractility, vascular tone, stroke volume variation, and related hemodynamic features before a conventional threshold is crossed. Because these measurements are dense, continuous, and temporally close to the event, they provide more immediate information than static demographic or comorbidity variables. The decline in performance at longer prediction horizons is therefore expected: the further the model is from the event, the less developed the physiological precursor pattern and the greater the opportunity for fluid administration, anesthetic adjustment, surgical stimulation, bleeding, positioning, or vasopressor treatment to alter the trajectory. However, temporal proximity can also inflate apparent discrimination because data immediately preceding hypotension may represent the early phase of the same physiological event rather than a genuinely distant forecast.

Across all outcomes, the high risk of bias identified in the analysis domain provides an additional explanation for apparently favorable model performance. Random splitting of records from one institution does not test whether a model generalizes across hospitals, calendar periods, devices, clinicians, coding practices, or patient populations. Hyperparameter tuning, feature selection, imputation, and oversampling must be conducted entirely within the training data; otherwise, information from the test set can indirectly influence model development. Failure to report calibration is also consequential. A model can rank patients correctly and therefore have high AUC while systematically overestimating or underestimating absolute risk. Such a model may be unsuitable for deciding whether prophylaxis, invasive monitoring, ICU admission, or intensified surveillance is warranted. Current guidance on perioperative risk prediction accordingly stresses that discrimination should be interpreted alongside calibration, threshold-specific performance, and decision-analytic measures [17,35,39].

Strengths and Limitations

This review has several strengths. It used a comprehensive multi-database search strategy incorporating controlled vocabulary and an extensive range of AI, machine-learning, perioperative, and outcome-specific terms. The review considered both established algorithms and contemporary ensemble and deep-learning approaches, reducing the risk that eligible studies were missed because they did not use the generic term “artificial intelligence.” Eligibility was restricted to the clinically coherent context of general anesthesia, while the outcome framework captured both common recovery-related events and serious physiological or prognostic outcomes. Study and model characteristics were extracted separately, allowing multiple algorithms from the same cohort to be described without treating each as an independent study. Performance was synthesized using AUC, sensitivity, and specificity rather than relying on a single metric, and findings were organized by outcome, predictor timing, model family, and clinical setting. The use of PRISMA 2020, TRIPOD-SRMA-informed extraction, and PROBAST+AI strengthened the transparency and methodological relevance of the review.

Several limitations should be acknowledged. First, the review covered five prespecified outcomes and should not be interpreted as a comprehensive assessment of all perioperative AI applications. Second, most included evidence was retrospective and derived from single institutions or selected surgical cohorts. Consequently, this review largely reflects reported model performance rather than prospectively demonstrated effectiveness. Additionally, models derived from single institutions may capture institution-specific patterns in clinical management and documentation, reflecting local clinical workflows rather than universally generalizable risk signals. Third, outcome definitions and ascertainment methods were inconsistent. This was particularly relevant to PONV, hypotension, neurological complications, and prolonged ICU stay, for which differences in assessment windows or institutional practice can substantially influence event classification. Finally, because meta-analysis was not performed, the review cannot provide a pooled estimate or formal statistical comparison among model classes. This limitation is appropriate to the evidence but restricts quantitative conclusions.

Implications for Clinical Practice

The findings do not currently justify routine, uncritical deployment of any identified model across general surgical populations. AI predictions should be regarded as decision-support information rather than replacements for anesthesiologist judgment. Before local implementation, the intended clinical use must be explicit: identifying patients for antiemetic prophylaxis is different from forecasting hypotension minutes before onset, selecting postoperative monitoring intensity, or reserving an ICU bed. Each application requires a defined prediction time, clinically meaningful threshold, corresponding intervention, and assessment of the harms of false-positive and false-negative classifications.

For PONV, AI could potentially complement established scores by incorporating procedure-specific and dynamic perioperative information. However, a model should be available before the prophylactic decision it is intended to guide and should demonstrate added value over a simple risk score. A highly sensitive but poorly specific model may lead to widespread additional prophylaxis, while a highly specific but insensitive model may miss many symptomatic patients. The preferred operating threshold should therefore reflect treatment safety, patient preferences, baseline risk, and the consequences of undertreatment rather than being selected solely to maximize a statistical index.

For hypotension, continuous waveform-based prediction represents the most immediately actionable application identified. A warning several minutes before threshold-defined hypotension could prompt evaluation of anesthetic depth, preload, vascular tone, blood loss, positioning, and impending surgical events. Nevertheless, alerts should be linked to a structured hemodynamic assessment rather than an automatic uniform treatment. Excessive fluid or vasopressor use may itself be harmful, and alarm fatigue could reduce responsiveness. Evidence that HPI-supported management reduces hypotension exposure is encouraging, but the limited and inconsistent evidence for improvement in downstream clinical outcomes warrants measured implementation with prospective audit [36-38].

For mortality, ICU utilization, and neurological complications, the most defensible near-term role is enhanced risk stratification and surveillance. Predictions might inform postoperative destination, senior review, handover, escalation planning, and the intensity of monitoring. However, risk estimate is valuable only if clinicians can identify an effective and proportionate response. Human-factors design is also essential. The Perioperative ORACLE randomized trial found that displaying machine-learning predictions influenced clinicians’ estimates but did not significantly improve their overall predictive performance, demonstrating that presenting an accurate model output does not automatically improve decision-making [35].

Recommendations for Future Research

Future studies should begin with a clearly specified clinical question rather than with the availability of dataset. Investigators should define the target population, intended user, exact outcome, prediction time, prediction horizon, and action expected after a high-risk result. Predictors must be available at the moment the prediction is intended to be used. Models that include postoperative pain, treatment administration, or early manifestations of an outcome should not be presented as preoperative prediction tools. Outcome definitions should be standardized and clinically meaningful, with separate modelling of mechanistically distinct events where possible.

External validation should be prioritized over the development of additional single-center models. Validation cohorts should differ geographically, temporally, and operationally from the development data and should include hospitals with different resources, clinical workflows, and diverse patient populations. The field must also progress from statistical validation to clinical-impact evaluation. Prospective silent deployment can first determine data availability, calibration drift, alert frequency, and workflow compatibility. This should be followed by randomized or carefully designed pragmatic evaluations comparing AI-supported pathways with usual care. Outcomes should include not only predicted events but also treatment changes, alert burden, unintended consequences, patient-centered outcomes, resource use, clinician trust, and cost-effectiveness. The potential for equitable distribution of clinical benefits following model implementation should also be assessed. Co-design with anesthesiologists, surgeons, nurses, patients, data scientists, and informatics teams will be essential to ensure that model outputs are understandable and connected to feasible action.

## Conclusions

AI models demonstrated potential for predicting PONV, perioperative hypotension, prolonged ICU stay, in-hospital mortality, and neurological complications following general anesthesia. The strongest performance was observed for short-horizon waveform-based hypotension prediction and selected procedure-specific or data-rich perioperative models. Nevertheless, performance varied markedly across populations and algorithms, and high discrimination did not consistently correspond to adequate sensitivity, specificity, calibration, or clinical utility. AI should therefore be considered a promising adjunct to perioperative risk assessment rather than a clinically established replacement for conventional assessment.
